# Personalizing neoadjuvant chemotherapy regimens for triple-negative breast cancer using a biology-based digital twin

**DOI:** 10.1038/s41540-025-00531-z

**Published:** 2025-05-23

**Authors:** Chase Christenson, Chengyue Wu, David A. Hormuth, Jingfei Ma, Clinton Yam, Gaiane M. Rauch, Thomas E. Yankeelov

**Affiliations:** 1 Departments of Biomedical Engineering, The University of Texas at Austin, Austin, TX USA; 2 Oden Institute for Computational Engineering and Sciences, The University of Texas at Austin, Austin, TX USA; 3 Departments of Imaging Physics, The University of Texas M.D. Anderson Cancer Center, Houston, TX USA; 4 Departments of Breast Imaging, The University of Texas M.D. Anderson Cancer Center, Houston, TX USA; 5 Biostatistics, The University of Texas M.D. Anderson Cancer Center, Houston, TX USA; 6 Institute for Data Science in Oncology, The University of Texas M.D. Anderson Cancer Center, Houston, TX USA; 7 Livestrong Cancer Institutes, The University of Texas at Austin, Austin, TX USA; 8 Departments of Breast Medical Oncology, The University of Texas M.D. Anderson Cancer Center, Houston, TX USA; 9 Departments of Abdominal Imaging, The University of Texas M.D. Anderson Cancer Center, Houston, TX USA; 10 Departments of Diagnostic Medicine, The University of Texas at Austin, Austin, TX USA; 11 Departments of Oncology, The University of Texas at Austin, Austin, TX USA

**Keywords:** Oncology, Mathematics and computing, Applied mathematics, Computational science, Computational biology and bioinformatics, Software

## Abstract

Despite advances triple negative breast cancer treatment, ~50% of patients will not achieve a pathological complete response prior to surgery with standard of care neoadjuvant therapy (NAT). We hypothesize that personalized regimens for NAT could significantly improve patient outcomes, which we address with a patient-specific digital twin framework. This framework is established by calibrating a biology-based model to longitudinal magnetic resonance images with approximate Bayesian computation. We then apply optimal control theory to either (1) reduce the final tumor cell number with equivalent dose, or (2) reduce the total dose of NAT with equivalent response. For (1), the personalized regimens (*n* = 50) achieved a median (range) reduction in the final tumor cell number of 17.62% (0.00–37.36%). For (2), the personalized regimens achieved a median reduction in dose delivered of 12.62% (0.00–56.55%) when compared to the standard-of-care regimen, while providing statistically equivalent tumor control.

## Introduction

Breast cancer is the most frequently diagnosed cancer in women worldwide with over 2 million new cases per year, leading to over 600,000 annual deaths^1^. In the United States, the death rate of breast cancer has decreased by 43% since 1990, which is attributed to advancements in targeted therapies depending on molecular subtypes of the cancer and increases in early detection^2^. However, these treatment advances have little effect on triple-negative breast cancer (TNBC) which is characterized by lack of expression of the progesterone, estrogen, human epidermal growth factor 2 receptors. TNBC represents 10–20% of all breast cancer cases^3,4^ and is primarily treated with neoadjuvant chemotherapy (NAT) followed by surgery^5,6^. More recently, immune check point inhibitors was approved by FDA for use in TNBC^7^, improving pathological complete response (pCR, i.e., no residual disease at the end of therapy) rates from 51.2% to 64.7%^8^. However, many patients still do not achieve pCR and there is still a definite need for improvement in the treatment and management of TNBC.

Despite classification as TNBC through molecular profling, the tumors in this class present with high levels of inter- and intra-patient heterogeneity, for example, through differences in genetic mutations and protein expression levels^9,10^. While NAT regimens are determined based on historical data from TNBC populations, others have suggested that personalized treatments may be necessary to target this heterogeneity^11,12^. We explore personalized therapy through adjustments to the scheduled chemotherapy regimen. Patients who would achieve pCR with the standard-of-care (SOC) regimen, as well as those who do not, could benefit from this personalized scheduling. For responders, providing a regimen with reduced doses without losing pCR could reduce the incidence of adverse events. For non-responders, personalized regimens have the potential to improve the probability of response prior to surgery.

A potential method for solving this personalization problem is through the use of patient-specific digital twins. A digital twin is defined by the National Academies of Sciences, Engineering, and Medicine (NASEM) as follows, “A digital twin is a set of virtual information constructs that mimics the structure, context, and behavior of a natural, engineered, or social system (or system-of-systems), is dynamically updated with data from its physical twin, has a predictive capability, and informs decisions that realize value. The bidirectional interaction between the virtual and the physical is central to the digital twin.”^13^. The digital twin framework can be combined with optimal control theory (OCT) to identify control inputs that maximize (or minimize) rewards for an individual patient, providing feedback to the physical space.

One of the earliest examples applying OCT to personalize chemotherapy regimens based on mathematical modeling is presented by Martin et al.^14^. The optimization problem was presented as a tradeoff between tumor response and accumulated drug concentrations. This idea of OCT and mathematical modeling has been expanded in recent years with increases in model complexity and advancements in optimization techniques^15–18^. However, to the best of our knowledge there is only one example where the mathematical modeling-informed treatment planning being tested in clinical settings is the adaption of dose-dense NAT based on the assumptions within the Norton-Simon hypothesis (which suggests that response rate is directly proportional to the tumor growth rate)^19^. We posit that the disconnect between model and clinic is in part due due to the limitation of calibrating complex models directly to measurable data. This has been addressed in recent years by validating predictions made by mathematical models parameterized directly by experimental^20,21^ or clinical data^22,23^. These models have now matured to the point where they can offer accurate spatio-temporal predictions of how tumors grow and respond to various therapeutic regimens and are therefore appropriate for the application of OCT. We expand on the examples provided by developing a framework based on personalized mathematical models that can be optimized under assumptions of uncertainty.

There has been limited success in finding optimal dosing schedules for individual patients based on mathematical model predictions and clincal data. This includes both trial and error approaches^24–26^ as well as optimal control problems^27,28^. There are several issues present in the existing literature on optimizing dosing scheduels that have prevented their wider adoption for identifying patient-specific regimens. First, the in silico regimens under consideration must be clinically viable. The problem is that clinically approved regimens are based on population data from the average patient, not allowing for deviations^29^. We address this through constraints related to drug concentration and toxicity, ensuring the computer generated regimens provide an equivalent or better risk profile based on quantifiable metrics as compared with the standard of care. Secondly, the computational load associated with high dimensional simulations can prevent evaluating new protocols in a clinically-relevant time frame. We employ reduced order modeling (ROM) methods to overcome this issue. Lastly, all mathematical models have an associated uncertainty that we seek to quantify to determine significance of the personalized predictions, moving away from a deterministic approach to personalizing therapy.

In this study we employ a partial differential equations (PDE) model to capture the spatio-temporal dynamics of tumor growth and treatment response. While ordinary differential equations can only capture bulk changes in the tumor over time, PDEs can successfully capture and predict the heterogeneity within a given patient^24,30^. Unfortunately, the simulation time increases with incorporation of spatial data; thus, three-dimensional PDEs with high resolution are notoriously computationally expensive which becomes a limiting factor when the goal is to inform and, even, guide clinical decision making. To address this issue, we have developed a framework for building a patient-specific ROM that is capable of reducing the inference and simulations times by factors of up to 1000 compared to a full resolution model^31^. This method is based on the proper orthogonal decomposition (POD), which finds a low dimensional space where model simulations can occur rapidly^32–35^. With the computational barrier removed, we have the opportunity to comprehensively investigate optimization of therapeutic regimens under uncertainty. First, Bayesian inference provides a way to assign probability distributions to model predictions^35,36^ for a given theoretical NAT regimen, but previously had limited applications with PDE approaches due to the computational burden. This probabilistic approach is beneficial as it allows for direct quantification of model uncertainty. Secondly, we can use the probabilistic spatio-temporally defined predictions to inform an optimal control problem for each patient. The application of ROM allows for efficient searching of an objective space, quickly testing NAT regimens to find the protocol that minimizes the pre-determined goal.

**Fig. 1:**
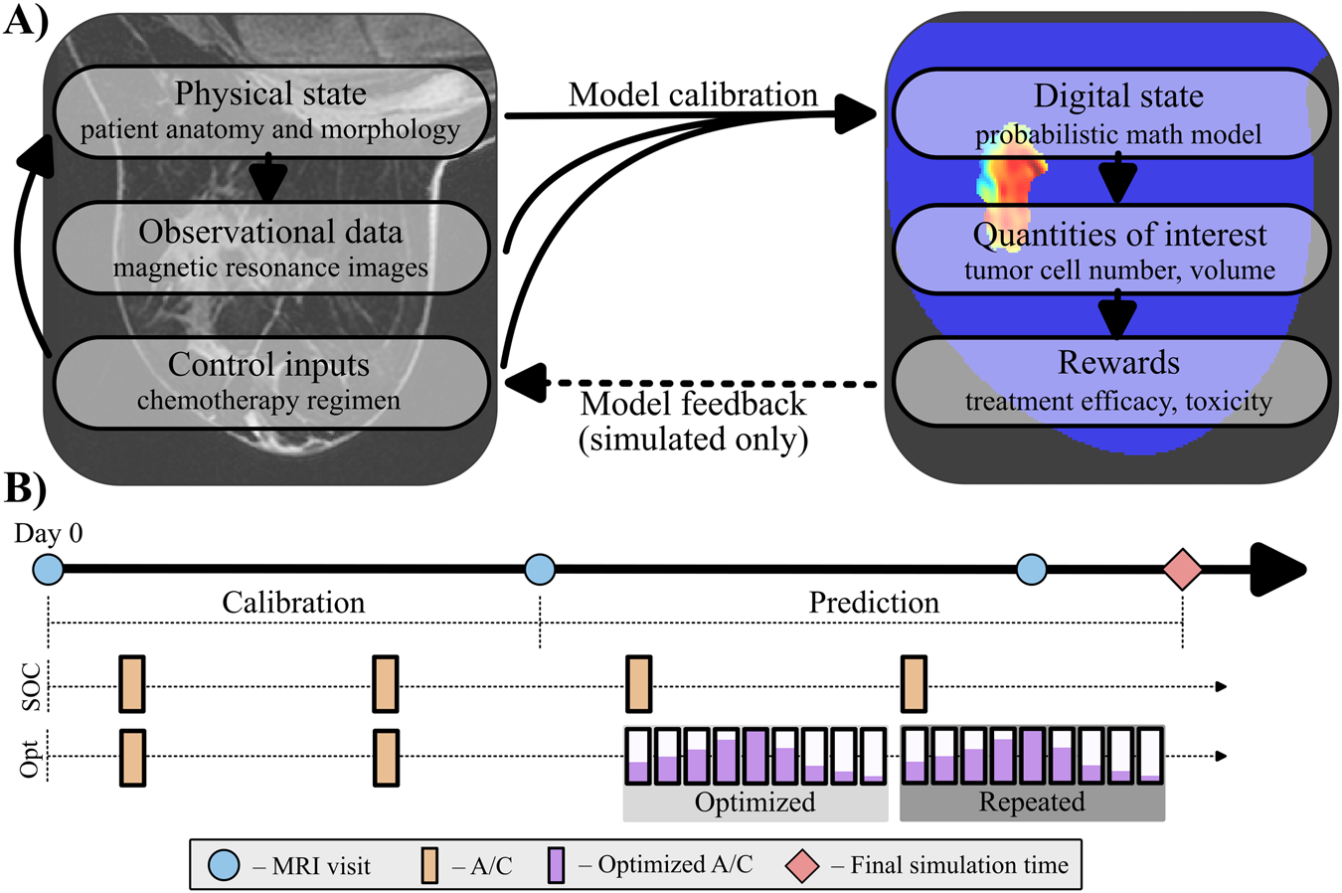
Digital twin overview. **A** Describes the format of the digital twin which consists of both physical and digital components. The physical component informs the status of its digital counterpart based on inputs from observational data and physically delivered controls (e.g., chemotherapy). The digital state is calibrated based on the physical components, then returns quantities of interest to derive or optimize associated rewards. The rewards can then be used as feedback to inform the next set of controls in the physical space. We provide personalized regimens that theoretically provide improved response based on simulation results. **B** Provides an overview of the clinical and modeling timeline. The physical space is represented by the blue circles (imaging) and orange rectangles (A/C treatment). The first two visits (V1 and V2) are used to calibrate the digital state, which can predict rewards at the end of the simulation. The rewards are optimized (e.g., minimized in the problems described) by adjusting the treatment schedule for the third and fourth cycles, consisting of one cycle of unknown variables (purple rectangles) which are delivered twice, sequentially.

In this effort, we present a framework for constructing digital twins (presented in Fig. 1) for TNBC patients that can be utilized to optimize NAT regimens on a patient-specific basis. This approach explicitly incorporates ROM and approximate Bayesian inference to reduce the computational burden of the problem, and quantify the uncertainty within the model calibrations, respectively. We apply this framework within an in silico study to identify its theoretical potential for personalizing NAT for TNBC patients based.

## Results

### Bayesian model calibration

**Fig. 2:**
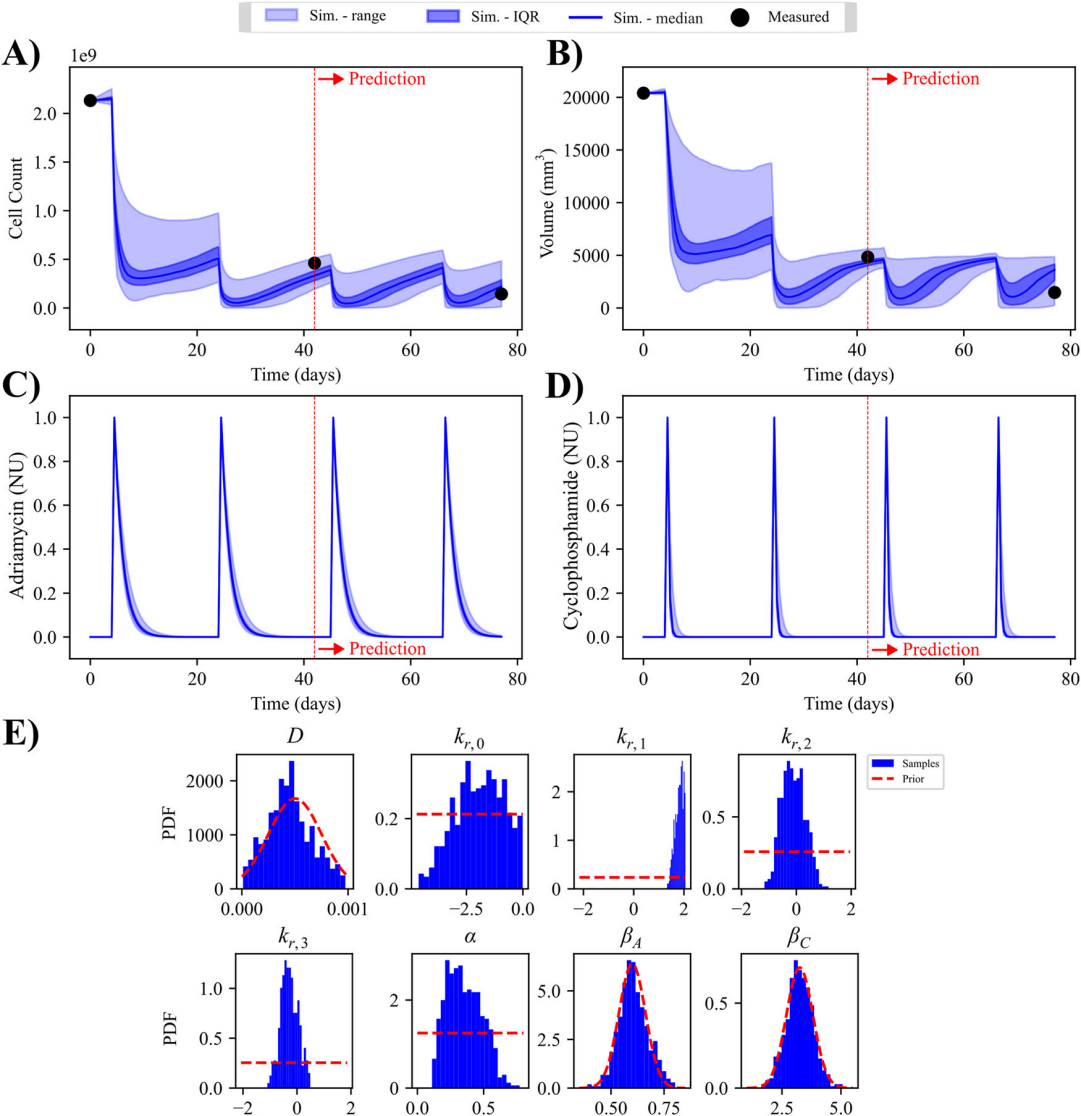
Patient-specific Bayesian calibration and prediction. **A** Presents the cell and **B** present the volume time courses for an individual patient. The model can successfully match the measured data at V2 with quantifiable uncertainty and make a prediction for V3. The drug curves in (**C**, **D**) show the concentration time course for the slow decaying Adriamycin (left) and fast decaying cyclophosphamide (right). In (**E**) parameter outputs from the approximate Bayesian computation are provided as probability distribution functions (PDF). Parameters, *D* (diffusivity), *β*_*A*_ (Adriamycin decay), and *β*_*C*_ (cyclophosphamide decay) sample directly from the prior (e.g., no deviation from prior based on measured data), whereas the *k*_*r,i*_ (reduced proliferation coefficients) and *α* (treatment efficacy rate) find an optimal distribution other than uniform prior (e.g., parameters move to patient-specific value based on measured data). The mathematical model fits the data well based on prior information, with uncertainty provided by likelihood of parameter fits.

Simulations with the calibrated digital twin provide an estimation of uncertainty over time based on the model fit and parameter assumptions through the approximate Bayesian Computation (ABC) method. In Fig. 2, an example simulation of the digital twin is presented for a median patient according to the percent error in both cell numbers and volumes at the calibrated and predicted time points (see Supplementary Figs. 1 and 2). In this case, after assimilation of the data from V2, the simulation displays a median (IQR) count of 3.32 × 10^8^ (2.81 × 10^8^, 3.81 × 10^8^) cells and volume of 4.32 (4.00, 4.54) mL, compared to a measured count of 4.61 × 10^8^ cells and volume 4.83 mL. At the V3 prediction time point, the model displays a median of 2.21 × 10^8^ (1.47 × 10^8^, 2.89 × 10^8^) cells compared to a measured 1.44 × 10^8^ cells. Similarly, the predicted volumes have a median of 3.61 (2.66, 4.15) mL compared to the measured V3 volume of 1.47 mL. The drug time courses in panels C and D of Fig. 2 show little variation (average range = 0.09 for Adriamycin, 0.024 for cyclophosphamide) based on the provided priors for the *β* parameters. Details on convergence of the Bayesian inference problem are provided in Supplementary Fig. 3.

**Fig. 3:**
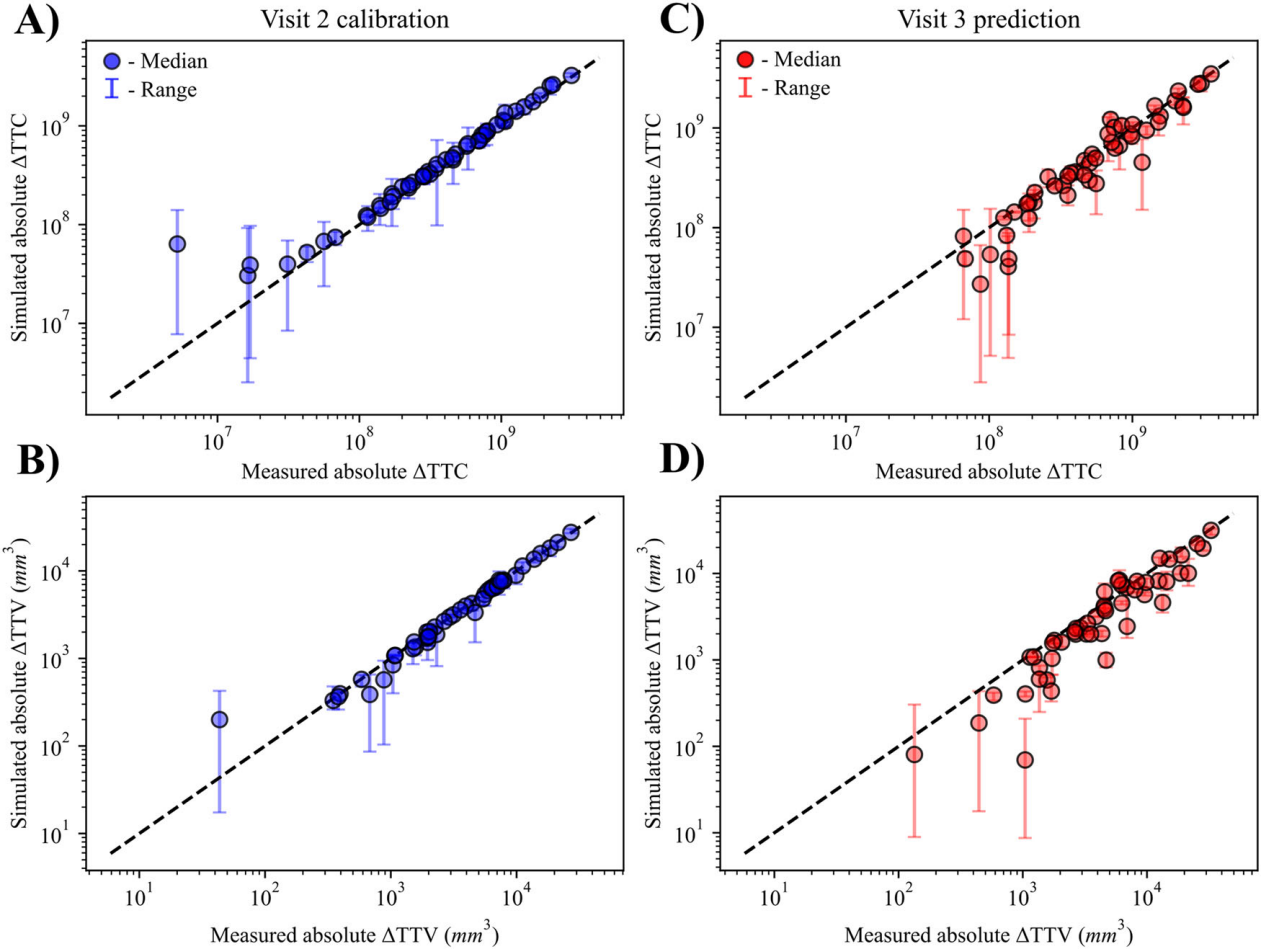
Cohort calibration results. **A**, **B** Provides correlations of the measured versus simulated cells and volume for the calibrated V2 time point across all patients. Despite the inclusion of uncertainty from the Bayesian inference, a high mean correlation is achieved for the cohort for the calibration (CCC = 0.99 for ΔTTC, CCC = 0.99 for ΔTTV). When predicting the ΔTTC and ΔTTV (**C**, **D**), the mean correlation decreases to CCC = 0.96 and CCC = 0.89, respectively.

Across the entire 50 patient cohort, there is a high correlation between the measurements and the model at both the V2 and V3 time points (see Fig. 3). To compare measurements and simulations, we calculate the absolute change in tumor cells (ΔTTC) or tumor volume (ΔTTV) from the baseline measurement. After the calibration, the CCC between the measured and simulated data at V2 are 0.98 (Standard Deviation (SD) = 0.00) and 0.99 (SD = 0.00) for the absolute ΔTTC and ΔTTV, respectively. The CCC between the measured and predicted absolute ΔTTC and absolute ΔTTV at V3 are 0.95 (SD = 0.01) and 0.89 (SD = 0.01), respectively. To compare the measured and simulated data on individual patients we calculate the percent error in the change from baseline for cells and volume. The median relative percent error of change in cell number is −8.90% (−16.81%, −2.47%) at V2 and 10.89% (0.00%, 30.69%) at V3 across all patients. For changes in volumes, the median percent error is 0.82% (−1.46%, 11.26%) at V2 and 22.03% (−69.85%, 48.73%) at V3. The spatially resolved maps are compared by computing the absolute percent difference between the simulated and measured maps in non-zero regions. The median absolute percent difference across all voxels and patients is 15.98% (8.33%, 22.28%) at V2 and 10.57% (3.72%, 43.41%) at V3. These prediction accuracies allow for increased confidence in subsequent in silico testing with alternative regimens, with results presented in Sections “Tumor burden minimization (P1)” and “Total chemotherapy minimization (P2)”.

### Risk assessment of treatment regimens

**Fig. 4:**
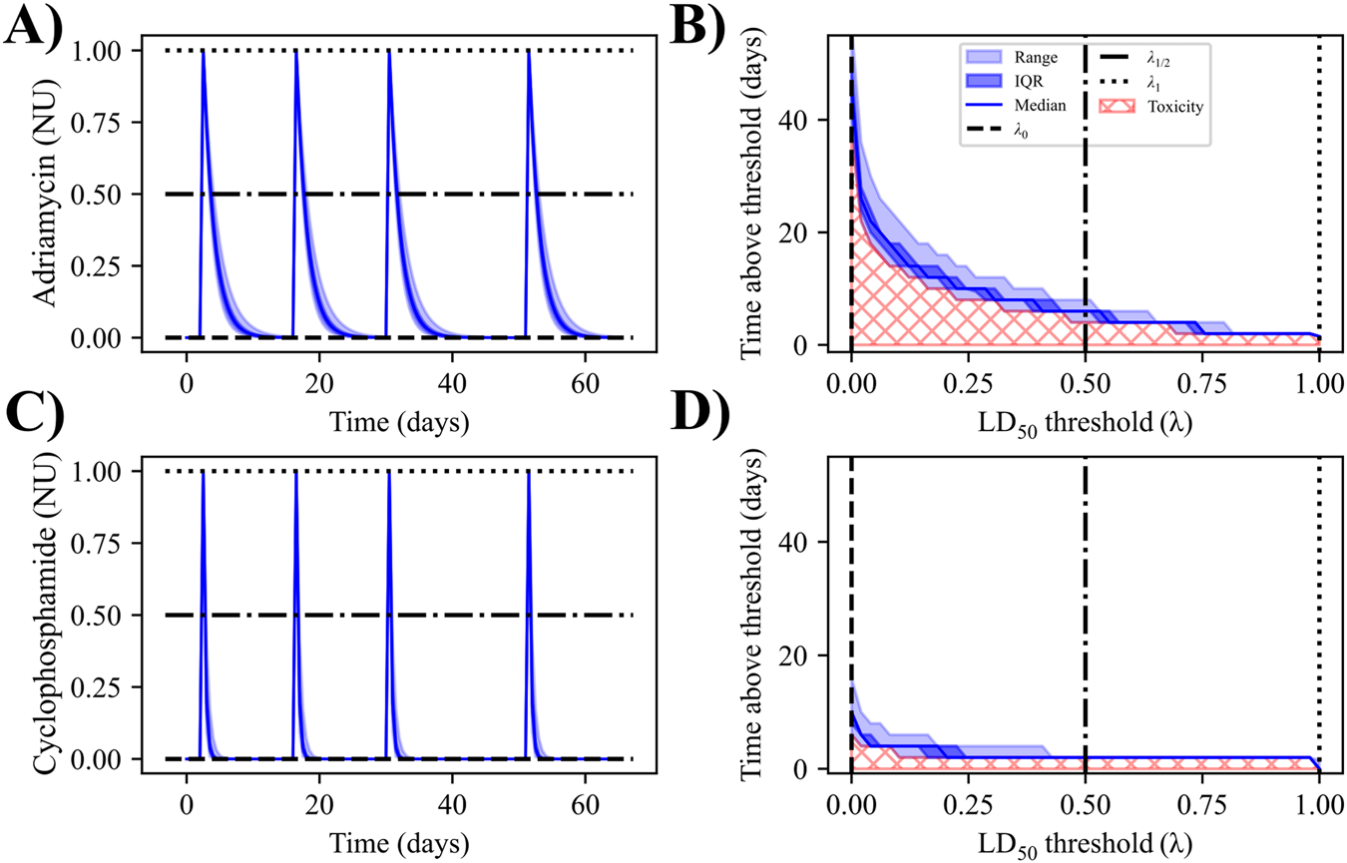
Toxicity estimation. In (**A**), the drug time course for Adriamycin is used to estimate the treatment regimen’s toxicity (**B**). The concentration time course is converted to a plot of the time above a given threshold based on the maximum concentration of the time course. The area under this curve (red hatching) becomes the estimate for toxicity. This toxicity estimate assumes that extended time above the LD_50_ threshold increases the risk associated with the regimen, evaluated with the assumption that the LD_50_ for each patient is unknown. The same process is shown in (**C**, **D**) for cyclophosphamide. Due to the faster decay, the toxicity due to cyclophosphamide is estimated to be less than Adriamycin.

Each treatment regimen provides a unique time course for each drug based on the days of delivery and the calibrated drug decay parameters, *β*_*j*_, that is used to determine the risk of the regimen. The drug time courses define the constraints and/or objectives depending on the optimization problem. Figure 4 provides example curves of drug concentration simulated with the digital twin assuming the SOC chemotherapy regimen that was actually delivered to the patient. (Note that each of the four doses of the SOC A/C regimen is merely 25% of the total (normalized) dose.) The maximum concentration is measured directly from the time course and is ~1.00 NU for both drugs. An example of the toxicity calculation (i.e., Eq. (14)) is seen in panels B and D of Fig. 4, where the toxicity for Adriamycin is 7.74 (SD = 0.79) TU and the toxicity for cyclophosphamide is 2.53 (SD = 0.25) TU. These values represent the baseline risk and an upper bound for what can be provided by the optimized protocol.

A comparison of the risk set for different potential regimens is provided in Table 1. Here we compare the four cycles of dose dense A/C therapy (4–AC–2) that was actually delivered to the patient to another clinically used option, four cycles of A/C therapy every 3 weeks (4–AC–3). In addition, two non-standard protocols are provided as examples of how the risk sets change with schedule. These regimens all deliver the MTD; thus, the normalized total dose of both drugs is equal to the number of cycles delivered. The maximum concentration also has the MTD as the upper bound for all simulated treatment regimens. The toxicity between the two SOC regimens (4-AC-2 and 4-AC-3) is not-significantly different for either Adriamycin or cyclophosphamide (*p* > 0.26). However, when comparing the 4–AC–2 regimen to a regimen with either two (2–AC–2) or six (6–AC–2) cycles of chemotherapy, the toxicity is now significantly different for both drugs (*p* < 0.001), with decreases in A/C toxicity for 2-AC-2 compared to 4-AC-2 and increases for 6-AC-2 compared to 4-AC-2.

**Table 1:** Risk associated with various A/C regimens

Treatment regimen	Adriamycin		Cyclophosphamide	
	Total dose (NU)	Toxicity (TU)	Total dose (NU)	Toxicity (TU)
*4–AC–2*	4	7.68 (SD = 0.78)	4	2.53 (SD = 0.24)
*4–AC–3*	4	7.67 (SD = 0.78)	4	2.53 (SD = 0.24)
*2–AC–2*	2	3.84 (SD = 0.39)	2	1.27 (SD = 0.12)
*6–AC–2*	6	11.52 (SD = 1.17)	6	3.80 (SD = 0.36)

Schedules are formatted as: (number of cycles)–(type of chemotherapy)–(weeks between cycles)

### Tumor burden minimization (P1)

**Fig. 5:**
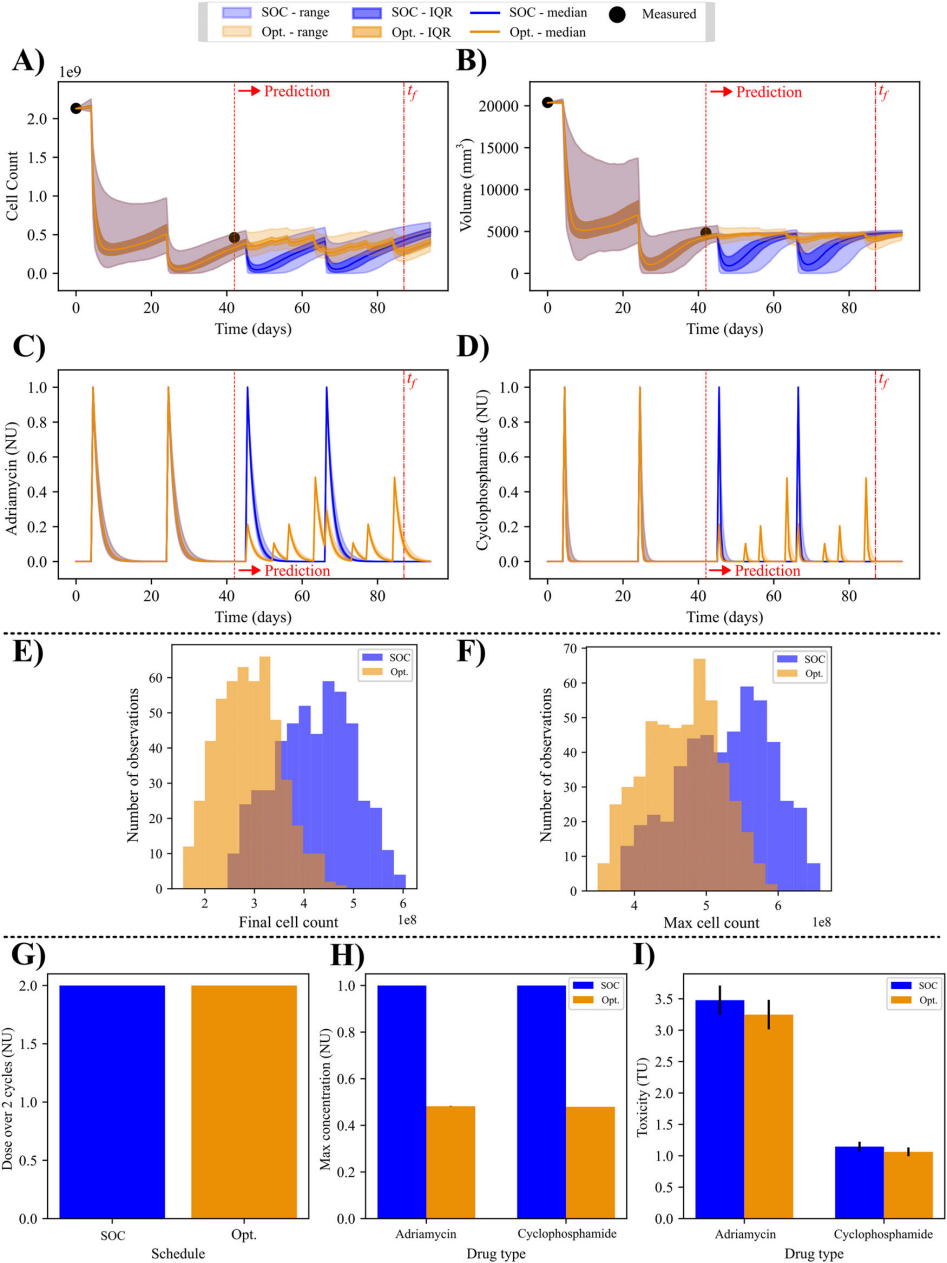
Patient-specific optimization of tumor burden with uncertainty. Results for the first optimization problem (P1, minimization of tumor burden) are shown for an example patient. In (**A**, **B**), the cell and volume time courses are provided for both the standard of care (blue) and optimized regimens (orange). The drug time courses in (**C**, **D**) show a more frequent delivery schedule after optimization, with three deliveries of varying chemotherapy dose per cycle, as opposed to a single dose at the maximum tolerated dose with the standard of care. **E** (final cell count) and **F** (maximum cell count) show the distributions of the two terms in Eq. (10) for the standard of care (blue) and optimized (orange) treatment regimens. A one-sided Mann-Whitney *U*-test shows a significant reduction in final cells when an optimized dosing schedule is followed (*p* < 0.001). **G**–**I** show the median (IQR marked if applicable) constraint values that are included in the optimization problem. These quantities of interest (i.e., total dose, max concentration, and toxicity) from the optimized regimen are approximately equivalent to, or less than the standard of care for both drugs. See Fig. 6 for the corresponding population results.

An example of the optimization for tumor burden is provided in Fig. 5. The optimized protocol consists of the original first two cycles of A/C therapy that was actually delivered to the patient, plus two additional cycles of an optimized regimen which, in this example, consisted of three deliveries of A/C per cycle. The new protocol results in a median percent change in cell number of −31.74% (−34.71%, −28.99%). Additionally, the new protocol results in a median (IQR) percent change in volume of −2.99% (−6.54%, −1.30%). Recall here that the optimization goal is not to reduce the maximum cell number, but rather to minimize total cell number at the end of the simulation. Despite this, there is a small percent reduction in the maximum cell count reached at any point during the simulation, with median = −11.80% (−12.45%, −10.84%). All three distributions, final cell number, final volume, and max cell number, exhibit significant reductions when simulating with the optimized regimens compared to the SOC regimen (*p* < 0.001). The total dose constraint displays a percent increase of 0.002% for the optimized protocol compared to the SOC, indicating a nearly identical total dose. The maximum concentration for the second half of the regimen (i.e., cycles three and four) has a median percent change of −51.80% for Adriamycin (*p* < 0.001) and −52.05% for cyclophosphamide (*p* < 0.001). The median percent change in toxicity is −7.15% (*p* < 0.001) and −7.30% (*p* < 0.001) for Adriamycin and cyclophosphamide, respectively.

**Fig. 6:**
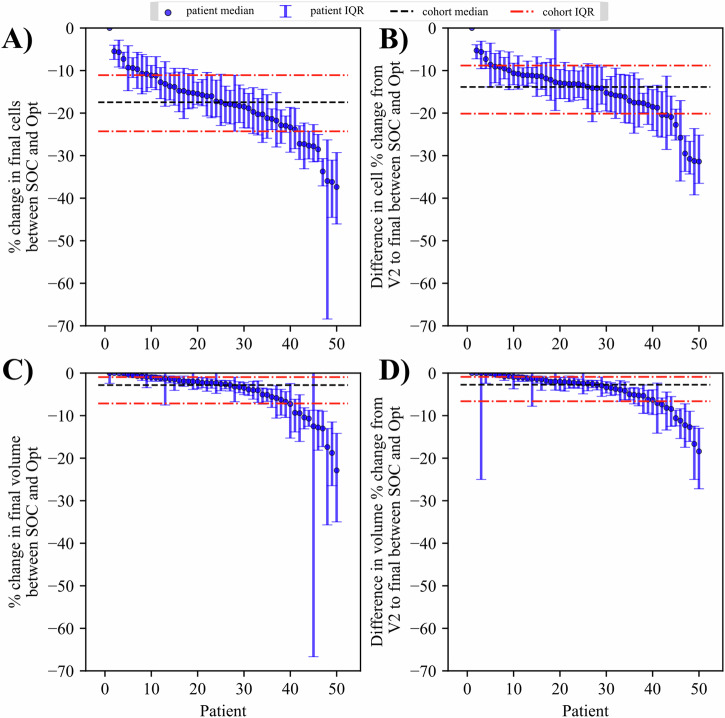
Cohort results from optimization of cell number. Results from all 50 patients in the cohort are presented, sorted from highest to lowest median of the quantity of interest (listed on vertical axis of each panel). In (**A**, **B**), the percent change between the standard-of-care and optimized results for final cells (**A**) and relative change in cells from V2 to the final time point (**B**) are presented. The final cells is our primary quantity of interest and has a median percent change of −17.62% for all patients; the reduction in final cells for individual patients is significant in 49 patients with *p* < 0.001. The relative change from V2 to the simulation end displays a median difference of -13.85% between the standard of care and optimized regimens and is significant in 49 patients with *p* < 0.001. For the tumor volumes in (**C**, **D**), we hope to see a corresponding negative change, or no change if negative is not possible. This holds true in all patients for the final volume prediction, with 49 patients displaying a significant reduction in volume (*p* < 0.001) and no change in 1 patient (*p* < 0.001) after optimization. The relative change in volume from V2 to simulation end is significantly better with optimization in 49 out of 50 patients with *p* < 0.001.

The results for the P1 optimization across all patients are provided in Fig. 6. For the entire cohort, the P1 optimization produced a median percent reduction of 17.62% (13.41%, 22.13%) compared to the SOC regimen (*p* < 0.001) for the predicted final cell count. In 49 out of 50 patients this reduction in final cell count was significant with *p* < 0.001, the only exception being the patient who saw no improvement. The difference in relative change from V2 to the end of simulation between SOC and optimized regimens is −13.86% (−20.13%, −8.81%) and is significant across the cohort at the 99.9% level. Additionally, while we do not optimize or constrain the tumor volumes in P1, we still see a median percent change of −2.82% (−7.14%, −0.94%) in the final volumes between SOC and optimized predictions. In 49 of 50 patients, the optimized regimen produced a significant reduction in volume compared to the SOC (*p* < 0.001). The relative change in volume from V2 to the end displays a median percent difference of −2.74% (−6.62%, −0.89%) between SOC and optimized regimens.

### Total chemotherapy minimization (P2)

**Fig. 7:**
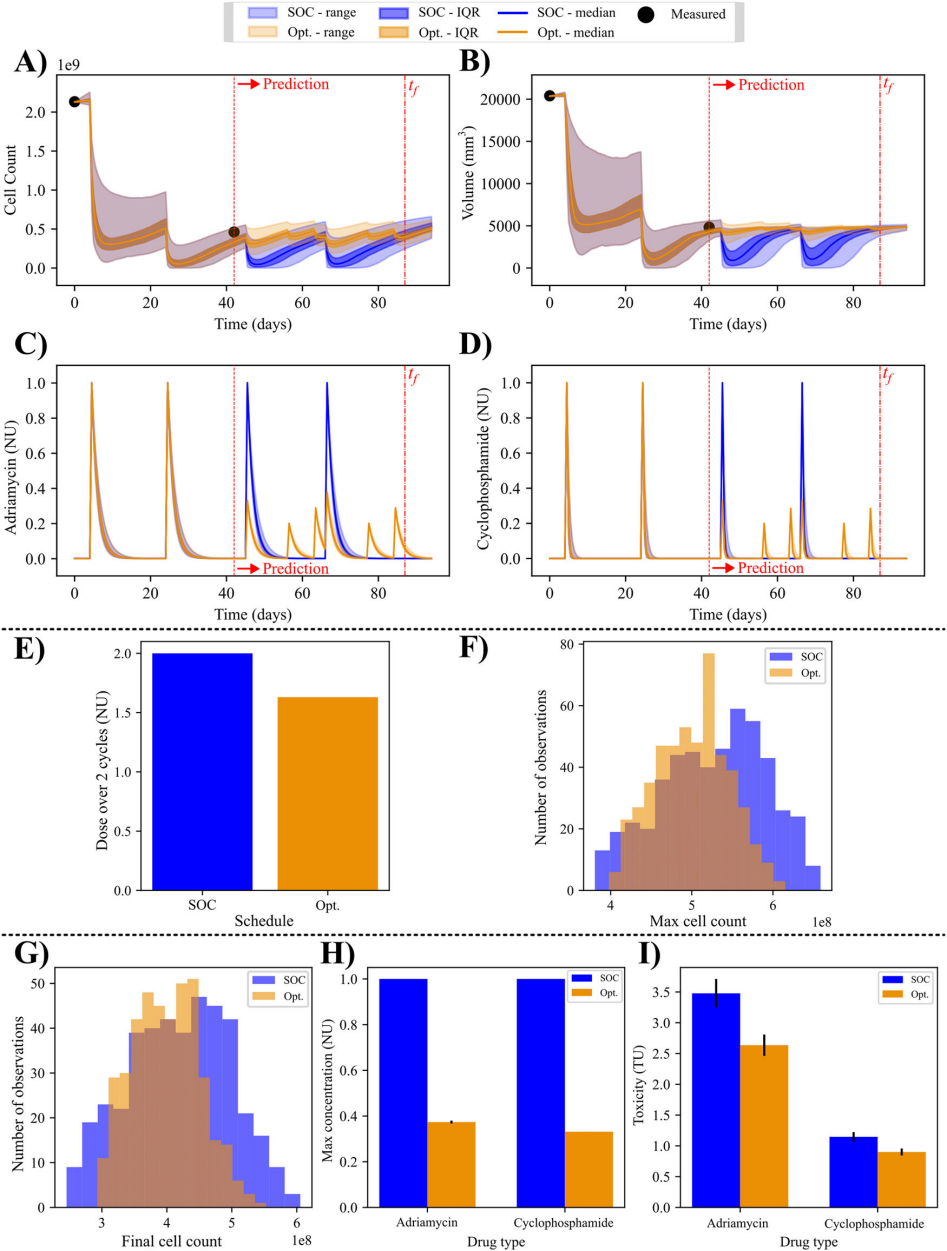
Patient-specific optimization of chemotherapy dosage with uncertainty. In (**A**, **B**), the time courses for the P2 optimization of an example patient are displayed. The cell and volume time courses show significant improvement for cells (*p* < 0.001) and no change for volumes (*p* = 0.17). The drug time courses in (**C**, **D**) show an optimal regimen consisting of three deliveries per cycle. The sum of these deliveries is visualized in (**E**) and is 1.63 normalized dose units compared to 2.00 for the standard of care. The constraints in (**G**–**I**) show that the final cells, maximum concentration, and toxicity estimation from the optimized protocol are equivalent to, or better than the standard-of-care. See Fig. 8 for the corresponding population results.

The second optimization problem has the goal of minimizing the total dose of chemotherapy while maintaining an equivalent response as the SOC. Results for the P2 optimization on the same patient depicted in Fig. 6 are shown in Fig. 7. In this patient, the optimization can achieve a significantly reduced final cell number (*p* < 0.001) while reducing the dose by 18.49% over the last two cycles. This reduction in total dose also provides concomitant reductions in the maximum concentration and toxicity with the optimized regimen. The maximum concentration has a median percent change of −62.65% (−63.04%, −62.23%) for Adriamycin (*p* < 0.001) and −66.88% (−66.87%, −66.89) for cyclophosphamide (*p* < 0.001). The estimation of the toxicity with the optimized schedule yields a median percent change of −24.92% (−25.57%, −24.42%) for Adriamycin (*p* < 0.001) and −22.94% (−24.42%, −22.06%) for cyclophosphamide (*p* < 0.001) when compared to the SOC regimen.

**Fig. 8:**
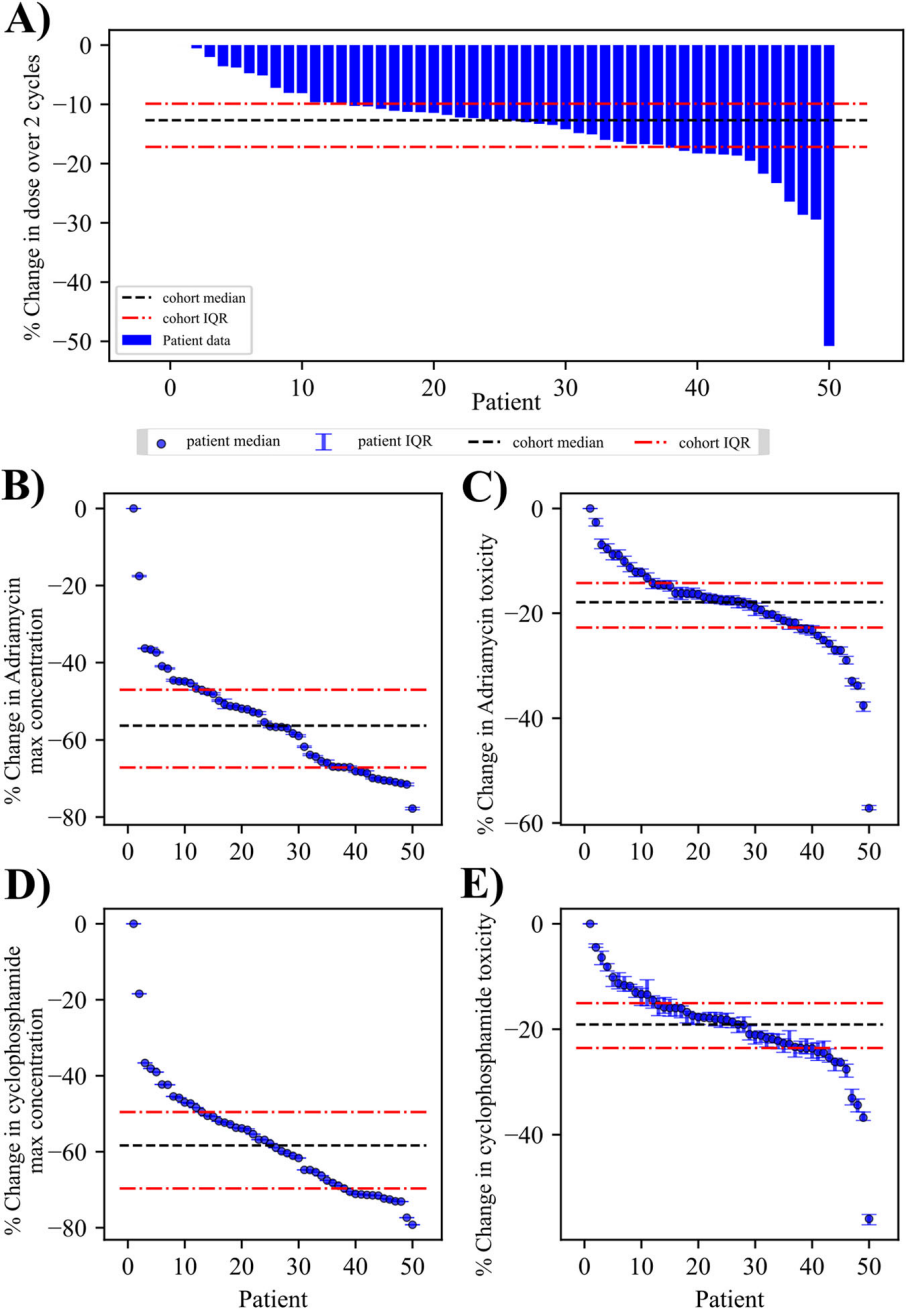
Cohort results from optimization of chemotherapy dosage. Results from each patient in the cohort for the chemotherapy optimization are displayed, sorted from highest to lowest for the respective metric indicated by the vertical axes. **A** Provides the percent change in total dose over the two optimized cycles compared to the standard of care. The method achieves a median reduction in dose of 12.68% (significant across cohort with *p* < 0.001). We additionally see reductions in the Adriamycin (**B**, **C**) and cyclophosphamide (**D**, **E**) estimates of maximum concentration and toxicity using the optimized regimen; significant reduction compared to maximum concentration and toxicity with standard of care in 49 patients for both drugs (*p* < 0.001).

The results for optimization problem P2 are shown in Fig. 8. Across all patients, the median dose required for the same response is 12.68% (9.90%, 17.18%) less than what was delivered for each patient during the final two cycles of SOC chemotherapy. As this optimal control problem assumes the drugs are delivered simultaneously, the maximum concentration and toxicity estimations had similar improvements compared to the SOC for both drugs. For the maximum concentration, the optimized protocols display a median percent change of −56.55% (−67.15%, −46.96%) and for Adriamycin and −58.40% (−69.67%, −49.55%) for cyclophosphamide. These changes were significant in 49 out of 50 patients (*p* < 0.001). The optimized plans also predict a significant reduction in toxicity in the same 49 patients (*p* < 0.001), with median percent changes in toxicity of −17.60% (−22.74%, −14.24%) for Adriamycin and −18.40% (−23.56%, −15.06%) for cyclophosphamide.

### Determining factors for optimization reward

**Fig. 9:**
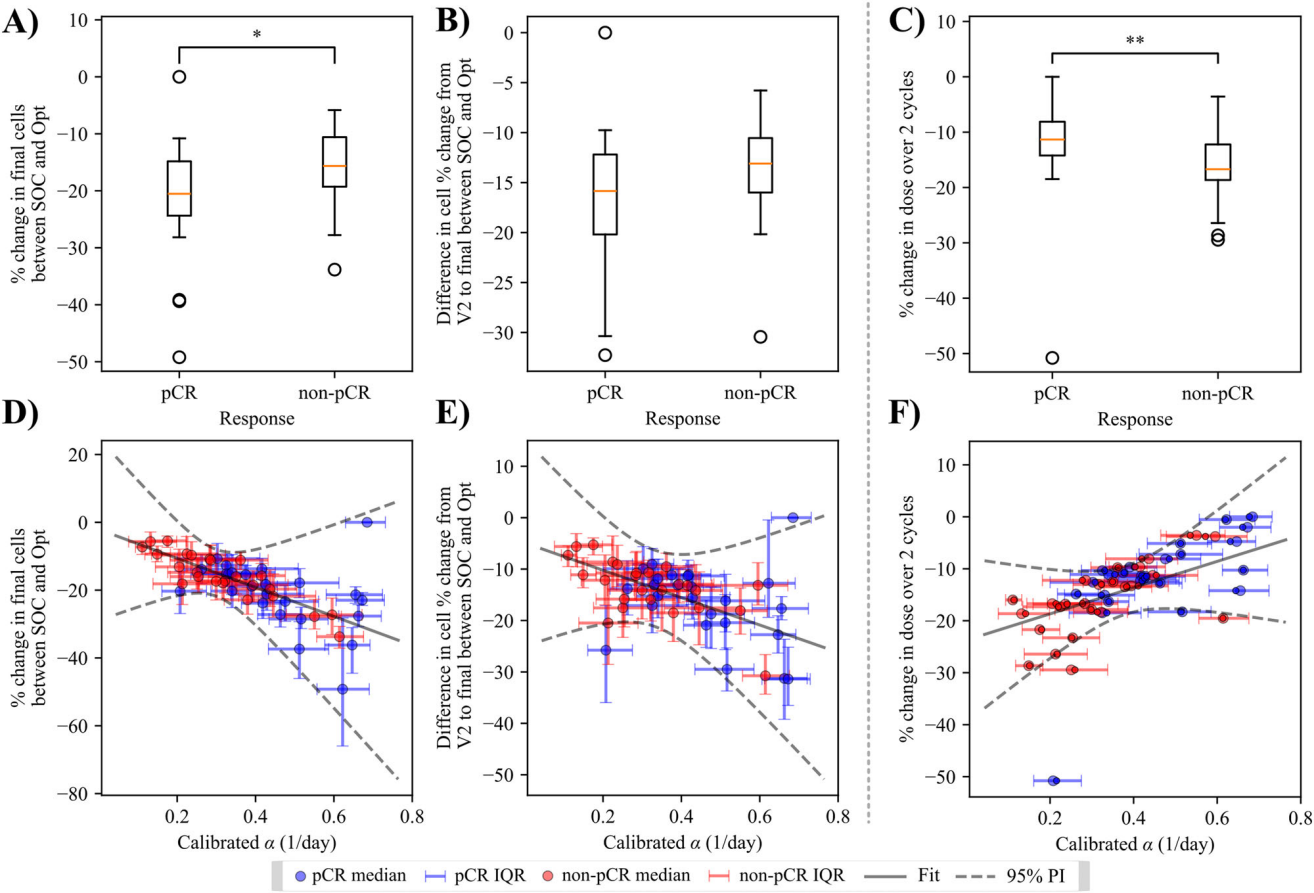
Clinical interpretation of optimization results. Results from the P1 and P2 optimization problems are presented for both pCR and non-PCR patients, along with plots against parameter fits for each quantity of interest. **A**, **D** Display results for the final cell quantity of interest (QOI). The optimization benefit according to percent change in final cells is significantly better for pCR patients at the 95% level (**A**). There is also a significant negative correlation in this QOI with calibrated alpha (Pearson *p* < 0.01.) (**B**, **E**) display the same analysis for the relative change in cells from V2 to end of simulation. When comparing this QOI with both clinical response (**B**), or calibrated alpha (**E**) there is no significant difference or correlation, respectively. **C**, **F** Display the results from the P2 optimization for percent change in dosage compared with clinical classification (**C**) and calibrated alpha (**F**). In (**C**), non-pCR show significantly better dose reduction with optimization compared to pCR patients (*p* < 0.01). In (**F**), a significant positive correlation (Pearson *p* < 0.05) is visible between dose change and calibrated alpha.

The patient dataset is equally split between patients who are classified as pCR and non-pCR after the completion of all therapy. (Note that patients received additional taxane and/or platinum based therapy post A/C; however, all simulations conclude following the A/C cycles). Optimization results for both P1 and P2 problems, separated by response classification are presented in Fig. 9. From the P1 problem, the percent change in final cells between the SOC and optimized predictions displays a median of −19.87% (−26.34%, −13.85%) for the pCR patients and −14.72% (−21.70%, −8.86%) for the non-pCR patients, and is significantly different with a *p* value of 0.033. These changes in final cells for individual patients are significantly correlated (*p* < 0.01) with the patient’s corresponding alpha value from the digital twin calibration. The difference in relative change in cells from V2 to the end of simulation between SOC and optimized is not significantly different for the clinical groups (*p* = 0.08) and is not correlated with the calibrated alpha values (*p* > 0.05). For the P2 results, the percent change in dose between SOC and optimized regimens is −11.33% (−14.22%, −8.11%) for pCR patients, and −16.70% (−18.64%, −12.22%) for non-pCR patients, with a significant difference at the 99% level (*p* = 0.0098). These dose changes exhibit a positive correlation with calibrated alphas (*p* < 0.05), opposite of the negative correlation seen with percent change in final cellularity in the P1 optimization.

## Discussion

We have developed a framework for building probabilistic digital twins for TNBC patients based on quantitative MRI and a biology-based mathematical model (i.e., Eqs. (2) and (3)). This framework takes advantage of a proper orthogonal decomposition-based ROM to provide high speed, spatially resolved simulations with uncertainty quantification through use of approximate Bayesian calibrations. Longitudinal MRI data representing spatial maps of tumor cellularity and drug concentration are used to calibrate the patient-specific model. The patient specific model is then used to make accurate predictions of tumor cellularity and volume at future time points. The calibrated model provides a high prediction accuracy across the cohort with CCCs of 0.84 ± 0.03 for cell numbers and 0.81 ± 0.01 for volumes when comparing model predictions and patient measurements.

After the model calibration phase, quantities of interest are informed based on distributions from the TNBC response prediction. We have shown that minimizing the rewards related to the quantities of interest can be framed as an optimal control problem to minimize either the tumor cellularity and volume or total dose administered. For the minimizing the tumor cellularity and volume problem (i.e., P1), the optimized regimen provides a median reduction in tumor cell number of −17.62% compared to the SOC regimen. In particular, the optimized distribution of predicted cell number at the simulation end is significantly better (*p* < 0.001) than the SOC in 98% of the patients. In the dose minimization problem, the model predicts no significant increases (*p* > 0.51) in tumor burden while using a median of 12.68% less dose than the SOC. These results could have a major impact on the design of future clinical trials seeking to optimize therapeutic regimens on a patient-specific basis. It is of note that the single patient who did not perform well with either optimization problem is the only patient with approximately zero cells at both V2 and V3. This limited the ability of the optimizer to move into new regions while the solver was running.

We also have identified components of the model that can imply the relative benefit of the optimization, prior to solving the problem. These features are directly related to tumor changes and parameters fit with the mathematical model. The results presented in Section “Determining factors for optimization reward” suggest that patients will have increased benefit in tumor cell minimization (problem P1) if they are pCR at the end of therapy compared to non-pCR patients. This finding stems from the fact that patients classified as pCR have lower tumor cellularity at V3 (median of 3.42 × 10^7^ cells for pCR vs. 2.45 × 10^8^ cells for non-pCR) and a similar absolute change in cell number results in a larger percent change. In the dose minimization (problem P2), the opposite is true, where non-pCR patients are more likely to benefit from dose reduction. In these cases, the assumption is that low calibrated *α* values prevent high levels of response to therapy no matter the treatment regimen, so larger reductions in total dose predict with equivalent total cellularity. These findings help elucidate optimization benefits based on tumor presentation.

While the SOC for TNBC (and, indeed, every other cancer) is currently based on historical response data from population-based studies, the present study indicates that a more individualized approach to treatment planning can positively benefit patients. It is important to note that the notion of personalizing therapy according to molecular profile is common practice in oncology. However, the notion of how to schedule and delivery such therapies has only recently been investigated^24,25^. By optimizing regimens based on a biology-based mathematical model, we can begin to optimize the delivery of the therapeutic regimens to target patient-specific differences and realize superior outcomes.

If we look further at the SOC for NAT, the MTD is found in all protocols, either in practice or in clinical trials. As this treatment approach provides only (approximately) a 50% pCR rate in TNBC patients, there is clearly still room to improve—especially given the high number of new breast cancer cases per year (~2 million worldwide^1^). A major result of the optimization in this work is that more frequent, smaller doses appear to provide an improvement in response, allowing for either smaller tumors at the end of NAT or reducing the total dose administered to the patient. The secondary benefit of the alternative regimens is decreased maximum drug concentrations for both Adriamycin and cyclophosphamide, potentially allowing for further reductions in chemotherapy-induced toxicities. In previous work, adverse events due to chemotherapy were reported in 26% of grade 1 and 2 tumors and 47% of those grades 3 and 4^37^. Studies focusing on cyclophosphamide found that reducing the dose of each delivery allowed for decreased toxicity and additional immune response^38,39^; importantly, low dose cyclophosphamide has not been evaluated to the same extent as therapy using the MTD in vivo. For Adriamycin, previous work suggests that patients receiving a continuous infusion, rather than bolus deliveries, achieved a similar tumor response but with a decrease in toxicity^40^. Being able to rigorously provide, *via* a biology-based mathematical model, a therapeutic regimen that achieves a similar—or better—response with decreased risk to the patient would represent an enormous step forward in the care of cancer patients.

It is important to stress that the digital twin framework has only identified alternative therapeutic regimens—they have not yet been tested or validated in a prospective trial. One focus of future validation studies should be on evaluating the toxicity of the optimized regimens. While we provide a quantification of the risk compared to the SOC, these are estimates based on data already collected or modeled. In particular, the toxicity estimation from the drug concentration curves makes assumptions on how extended periods of high chemotherapy concentrations will negatively impact the patient, which directly influences the optimization. Inclusion of a dedicated model for toxicity is of central importance but requires further data from individual patients to include in the calibration. This would allow for replacement of the toxicity estimation in Eq. (12), with a more biologically imformed risk for a given therapy. Testart-Paillet et al.^41^ provide a review of potential models for hematological toxicities from chemotherapy that could be integrated into the digital twin framework. Similarly, a model of white-blood cell counts^42^ could be included for each patient with the appropriate data.

Another set of opportunities for improvement of the framework are based on the model itself. While the digital twin calibration allows for a patient specific assessment of uncertainty in predictions, there are mechanisms the model does not capture. While the PDE successfully captures spatial heterogeneity of growth, it does not account for heterogeneity of efficacy. The intratumoral heterogeneity can prove difficult to treat^43^, as it has both a spatial and temporal component^44^ and needs to be addressed within the mathematical model. A second missing component is the development of resistance to chemotherapy^45^. Models have attempted to capture this behavior^46,47^, but can be difficult to parameterize with in vivo data. The primary concern is differentiating between sensitive, and resistant cells in a spatial model, requiring additional data related to intra-tumoral heterogeneity to accomplish this task. Inference for resistance development is possible^46^ but has limited application in vivo due to the quantity of longitudinal measurements required. Lastly, the model is only capable of predicting, and optimizing response, to therapies that have already been delivered to the patient; this limits the inclusion of new chemotherapies or immunotherapies in the framework. We do note that we have recently introduced a methodology that may allow for performing this optimization after the first imaging data set is acquired, but prior to initiation of NAT^48^.

We finally want to highlight that this optimization problem is not the only option for personalizing therapy in TNBC. We have formulated the two problems to focus on tumor burden minimization, and dose de-escalation, with constraints based on estimated quantities of interest from a drug decay model (population averaged protocols provided in Supplementary Fig. 4). These problems were constructed with additional weighting in the objective to prevent delaying drug delivery, but results in Supplementary Figs. 5 and 6 show that removing this weighting term results in significantly better optimization results. While the additional treatment plans provided in the supplemental might not be clinically relevant due to the additional tumor burden allowed, it shows that there are other plans that can outperform the standard of care. Additionally, the optimization objectives in both P1 and P2 are dependent on the time chosen for the simulation end point. This work tries to enforce a fair comparson between optimized and standard-of-care protocols by fixing the cycle length for each patient, but future work should seek to find personalized regimens that are independent of the optimal control problem. There are also opportunities for dose-escalation in patients where chemotherapy tolerance is expected to be high, as seen by reduced toxicities from the P1 optimization results. This would require additional validation of the optimized regimens with pre-clinical models to prevent unwanted side effects but shows that deviation from MTD therapy could allow for higher total doses to be delivered.

Utilizing biology-based mathematical models, proper orthogonsectiuon 4al decomposition, and approximate Bayesian inference, we have developed a framework for constructing digital twins to identify optimal interventional strategies for individual breast cancer patients. This methodology can predict response to A/C chemotherapy with high accuracy and quantified uncertainty, and provide guidance on tailoring the chemotherapy regimen to optimize patient outcomes. In particular, we provided a formalism that allows for either a reduction in tumor burden at the end of treatment, or reduced drug concentrations delivered to the patient, both of which could lead to improved quality of life for these patients.

## Methods

### Patient data

This study is a retrospective analysis of 50 TNBC patients enrolled in the clinical trial, “A Robust TNBC Evaluation Framework to Improve Survival,” (ARTEMIS, NCT02276443^49^). Patients who underwent standard anthracycline-based chemotherapy with Adriamycin and cyclophosphamide (A/C) are included. This study is only focused on the optimization of A/C scheduling, and therefore it does not include data on taxane or investigational drugs. Only the salient details of the trial are described below; a more complete description is available in refs. ^23,49^.

For each patient, magnetic resonance imaging (MRI) data was acquired at baseline (V1), post two cycles (V2), and post four cycles (V3) of A/C. Each session provided both diffusion-weighed (DW) MRI and dynamic contrast-enhanced (DCE) MRI, collected on a GE Discovery MR750 or MR750w whole-body scanner (GE Healthcare, Milwaukee, USA). The DW-MRI data was collected using a 2D spin echo echo planar imaging sequence^50^ on a matrix of 80 × 80 × 60 voxels, yielding a spatial resolution of 2 × 2 × 4 mm^3^. An apparent diffusion coefficient (ADC) map was subsequently derived on a GE AW server (v3.2; GE Healthcare) from the DW-MRI data. The DCE-MRI data was collected using a 3D differential subsampling with cartesian ordering (DISCO) sequence^51^ on a 320 × 320 × 140 voxel grid yielding a spatial resolution of 0.58 × 0.58 × 1.6 mm^3^. For the DCE-MRI scan, an initial set of images was acquired prior to injection of the contrast agent (Gadovist, Bayer HealthCare, Leverkusen Germany). Images were the acquired after contrast injection every 8–15 s (11 s median) with a range of 32–64 acquisitions depending on breast size. Tumor segmentations for each visit were supplied from radiologists based on manual segmentations using DCE-MRI data.

### Image processing

Here we describe only the salient points of the image processing, a full description of the pipeline can be found in refs. ^23,52^. All image processing was performed in MATLAB (R2021a; MathWorks, Natick, MA, USA^53^)

For each scanning session, all images were registered to a common space to reduce the effects of patient motion; this is *intra*-session registration. Briefly, a rigid 3D registration was performed to align the DCE and DW images. This is accomplished *via* MATLAB’s “*imregtform*”. Next, the *intra*-visit registered MR images from the three time points (see Fig. 1) are aligned using a non-rigid registration^54–56^, that maintains the segmented tumor volumes but allows for deformable breast boundaries. This inter-visit registration facilitates the modeling procedure described in subsequent Sections “Mathematical model for TNBC response”, “ROM derivation”, and “Bayesian parameter inference”. After registration, the outputs are downsampled by a factor of four in the in-plane directions to reduce the computational requirements of the problem.

Next, quantitative metrics defining tumor cellularity and drug concentration are derived from each visit’s scans. First, the ADC maps are used to estimate the local cellularity within the breast tissue based on inverse relationship with water mobility^57–59^ as seen in Eq. (1):

1 $$\hat{N}({\boldsymbol{x}},t)=\theta \left(\frac{AD{C}_{w}-ADC({\boldsymbol{x}},t)}{AD{C}_{w}-AD{C}_{\min }}\right),$$

where $\hat{N}({\boldsymbol{x}},t)$ are the measured cellularity maps from each visit (*t* ∈ [V1, V2, V3]), *θ* is the physical carrying capacity given the voxel and tumor cell sizes, *ADC*_*w*_ is the ADC of water^60^, *ADC*(***x***,*t*) is the voxel resolved ADC map from each visit, and *ADC*_min_ is the minimum ADC value found within the tumor segmentations across all visits. The carrying capacity is determined by assuming a cell size of 10 μmol/L^61^ and a packing density of 0.7405^62^. Outside of the tumor segmentation, $\hat{N}({\boldsymbol{x}},t)$ is set to zero.

Lastly, the DCE-MRI data is used to inform the simulation boundaries as well as the delivery and accumulation of the chemotherapies. The initial pre-contrast acquisition of the DCE-MRI is used to manually determine the edges of the breast, which was used to bound the tumor during simulation. The full spatio-temporal DCE-MRI acquisition is then used to estimate the local contribution of chemotherapy based on the area under the intensity curve (AUC) at each voxel. The AUC map is locally defined, and following its calculation is normalized to a scale of zero to one. This map does not directly simulate the active drug concentration but rather serves as an estimation for where chemotherapy is likely to be based on contrast permeability^63,64^. For further details on image processing and outputs please see ref. ^23^.

### Mathematical model for TNBC response

We use a biology-based mathematical model to simulate the spatio-temporal dynamics of breast tumors on a patient-specific basis. This model is calibrated directly to the imaging data described in the Section “Image processing”. The model is built on the Fisher-Kolmogorov equation which describes the invasion and growth of the tumor, with an additional term describing the response to chemotherapy as described in ref. ^23^ and presented in Eq. (2):

2 $$\frac{dN({\boldsymbol{x}},t)}{dt}=D\Delta N({\boldsymbol{x}},t)+k({\boldsymbol{x}})N({\boldsymbol{x}},t)(1-N({\boldsymbol{x}},t))-N({\boldsymbol{x}},t)\sum _{j}{\gamma }_{j}({\boldsymbol{x}},t).$$

In Eq. (2), N(***x***,*t*) represents the modeled tumor cellularity at position ***x*** and time *t*, *D* is the diffusivity coefficient, *k*(***x***) is a spatially resolved proliferation map and *γ*_*j*_ is a spatio-temporally resolved function describing the effects of the *j*th chemotherapy drug (where *j* ∈ [*A*, *C*], where “A” and “C” denote Adriamycin and Cytoxan, respectively). This treatment function is defined by Eq. (3):

3 $${\gamma }_{j}({\boldsymbol{x}},t)=\alpha \mathop{\sum }\limits_{k=1}^{{T}_{j}}AU{C}_{k}({\boldsymbol{x}}){C}_{j,k}H({C}_{j,k}-0.1){e}^{-{\beta }_{j}(t-{\tau }_{j,k})H(t-{\tau }_{j,k})},$$

where *α* is drug efficacy, *AUC*_*k*_(***x***) is the AUC of the spatio-temporal enhancement from the most recent DCE-MRI session to estimate the local drug distribution of the *k*th chemotherapy delivery, *T*_*j*_ is the total number of doses of drug *j*, *C*_*j,k*_ is the concentration of drug *j* at the *k*th delivery, *β*_*j*_ is the drug specific decay rate, *τ*_*j,k*_ is the time of the *k*th delivery of drug *j*, and *H* is the Heaviside step function. The first step function (i.e., following the summation) provides a threshold that *C*_*j,k*_ must surpass in order to be recognized as a delivery; if the threshold is not met, the summation is equal to zero for the specific values of *j* and *k*. The value of 0.1 in this function represents 10% of the maximum tolerated dose. The second step function (i.e., in the argument of the exponential term) serves as an “on” switch for each delivery based on the time of delivery. The spatially resolved model allows for estimating local distiributions of cellularity based on a computed distribution of chemotherapy. A summary of the model parameters and their assignment is presented in Table 2.

**Table 2:** Descriptions and assignment of model parameters and variables

Variable name	Description	Units	Assigned by
*N*(***x***,*t*)	Spatiotemporal cellularity	cells	DW-MRI (measured); simulation
*AUC*_*k*_(***x***)	Relative concentration map	dimensionless	Calculated from DCE-MRI
*τ*_*j,k*_	Drug injection times	day	Treatment protocol
*C*_*j,k*_	Drug concentration	dimensionless	Treatment protocol
*γ*_*j*_	Cell death rate	day^−1^	Eq. (3)
*D*	Diffusivity coefficient	mm^2^/day	Calibrated
*α*	Drug efficacy	day^−1^	Calibrated
*β*_*j*_	Drug decay rate	day^−1^	Calibrated
*k*(***x***)	Proliferation map	day^−1^	Calibrated

### ROM derivation

The ROM is built on the measured cell maps derived from DW-MRI, $\hat{N}$(***x***,*t*), using a POD based framework. While we summarize the important points of this model construction below, the interested reader is referred to ref. ^31^.

The goal of POD is to find a reduced space that reflects the details contained in the physical measurements. This is accomplished by finding the singular value decomposition (SVD) (performed with MATLAB’s “*svds*”) of $\hat{N}$(***x***,*t*). Data from timepoints V1 and V2 are used to construct a matrix of snapshots, *S*(***x***,*t*), augmented through a series of averaging and filtering to fill in the time gap between images, which approximates $\hat{N}$(***x***,*t*) over time. The decomposition of the snapshots then results in a set of coefficients, *ψ*_*i*_, and modes, *ϕ*_*i*_, so that:

4 $$S({\boldsymbol{x}},t)\approx \mathop{\sum }\limits_{i=1}^{r}{\psi }_{i}(t){\phi }_{i}({\boldsymbol{x}}),$$

where *r* is the number of modes required for the approximation to hold. The value of *r* is determined by the decay of the singular values so that the majority of the variance in the snapshot matrices (e.g., variance) is captured by the retained modes (conventionally 99.5% cumulative energy^32^). The retained modes form a projection basis, *V*, to be used in the POD.

To apply POD, Eq. (2), which defines the full order model, is formatted into a set of ordinary differential equations where each model term is represented by an operator (or two for the proliferation term) as in Eq. (5):

5 $$\begin{array}{ll}\frac{\partial N({\boldsymbol{x}},t)}{\partial t}=A(D)N({\boldsymbol{x}},t)+B(k({\boldsymbol{x}}))N({\boldsymbol{x}},t)-H(k({\boldsymbol{x}}))(N({\boldsymbol{x}},t)\otimes N({\boldsymbol{x}},t))\\\qquad\quad-\,\mathop{\sum }\limits_{j}\mathop{\sum }\limits_{k=1}^{{T}_{j}}{T}_{j}({\gamma }_{j}({\boldsymbol{x}},t))N({\boldsymbol{x}},t),\end{array}$$

where *A* represents the diffusivity operator determined by *D*, *B* and *H* represent proliferation operators controlled by *k*(***x***), and *T*_*j*_ represents drug specific treatment operators determined by *γ*_*j*_. The operators are implemented assuming 2nd order central differences, with zero-flux boundary conditions enforced along the edges of the breast segmentation. These operators are projected into the reduced space that minimizes the *l*_*2*_-norm of the snapshots with a Galerkin scheme^33,65^ using the previously determined basis *V*. Additionally, the local parameter *k*(***x***) found in Eqs. (2) and (5) is reduced with a Karhunen-Loeve decomposition^66^, forming the new parameter *k*_*r*_ based on the mapping of basis *V*. This reduction simplifies the number of parameters required for model personalization. These reductions result in the reduced form of Eq. (5):

6 $$\begin{array}{ll}\frac{\partial {N}_{r}(t)}{\partial t}={A}_{r}(D){N}_{r}(t)+{B}_{r}(k({\boldsymbol{x}})){N}_{r}(t)-{H}_{r}(k({\boldsymbol{x}}))({N}_{r}(t)\otimes {N}_{r}(t))\\\qquad\quad\;\;-\mathop{\sum}\limits_{j}\mathop{\sum }\limits_{k=1}^{{T}_{j}}{T}_{{j}_{r}}({\gamma }_{j}({\boldsymbol{x}},t)){N}_{r}(t)\end{array}$$

where *N*_*r*_(*t*) represents *N*(***x***,*t*) at any given time in the reduced space. The selection of *r* so that *r* « *n*, where *n* is the rank of the original problem in Eq. (5), ensures that solving Eq. (6) will require a reduction in computational time. Solving Eq. (6) requires application of a forward Euler time stepping to increment the values of *N*_*r*_(*t*) throughout time, with a time step chosen so that the maximum diffusivity of 1e-3 mm^2^/day is stable. It is of note that this method for constructing the ROM is intrusive, as such it requires modification to the code for the full order solver.

### Bayesian parameter inference

The personalization of the digital twin to a given patient is achieved by calibrating the model parameters to the imaging data measured from an individual patient. We utilize a Bayesian approach to provide a probabilistic prediction based on sampled sets of calibrated parameters, *θ*. For each patient and sample, *θ* consists of diffusivity rate *d*, reduced proliferation *k*_*r*_, drug efficacy *α*, and drug specific decay rates *β*_*j*_. Due to the non-normal error distributions provided by the ROM (see Supplementary Fig. 7), an approximate Bayesian computation (ABC)^67,68^ is used to sample the posterior distribution. In this formulation, the posterior is formed by sampling directly from the parameter priors, and accepting the samples if Eq. (7) holds true:

7 $$\rho \left({\hat{N}}_{r}(t),{N}_{r}(\theta ,t)\right)\le \varepsilon$$

where *ρ*(${\hat{N}}_{r}\left(t\right),\,{N}_{r}$(*θ*,*t*)) measures the distance between the observed and simulated data, and *ε* is a pre-defined tolerance limit. While the mean squared error (MSE) is used to represent *ρ*(${\hat{N}}_{r}\left(t\right),\,{N}_{r}$(*θ*,*t*)), it does not address the tolerance, *ε*. This value directly informs the probability with which to accept parameters and must be close to the true model uncertainty for each patient. At either of the extremes (i.e., too high or too low *ε*), the sampler will either accept all samples from the prior, or only accept perfect samples, both of which are undesired. To assign this tolerance we first find the parameters, *θ*_*LM*_, that minimize the *l*_*2*_-norm between the data and model at V2 with the Levenberg-Marquardt algorithm, using the framework outlined in ref. ^31^. The patient-specific tolerance is then set according to Eq. (8):

8 $$\varepsilon =\rho \left({\hat{N}}_{r}(t),{N}_{r}({\theta }_{LM},t)\right)$$

which allows for the sampler to find a distribution of parameter sets that exhibit a similar MSE to the least-squares minimized solution.

To solve for the posterior distribution, prior distributions are defined utilizing knowledge on valid ranges and parameter identifiability. The priors for each parameter are presented in Table 3.

**Table 3:** Parameter prior distributions

Variable name	Description	Distribution
*D*	Diffusivity rate	${\mathscr{T}}{\mathscr{N}}(0.5\times {10}^{-4},2.5\times {10}^{-4},{10}^{-6},{10}^{-3})$
*α*	Drug efficacy	${\mathscr{U}}({10}^{-6},1.0)$
*β*_*A*_	Adriamycin decay rate	${\mathscr{T}}{\mathscr{N}}(0.60,0.06,0.35,0.85)$
*β*_*C*_	Cyclophosphamide decay rate	${\mathscr{T}}{\mathscr{N}}(3.25,0.56,1.00,5.50)$
kr,i	Proliferation coefficient	${\mathscr{U}}({k}_{r,i,\,\min },{k}_{r,i,\,\max })$

*𝒯𝒩* Truncated normal distribution, *𝒰* Uniform distribution.

In Table 3, 𝒯𝒩(*μ*,*σ*^*2*^,*a*,*b*) refers to a truncated normal distribution with mean and standard deviation of *μ* and *σ*^*2*^, respectively, and truncation points at *a* and *b*. 𝒰(*a*,*b*) represents a uniform distribution with bounds at *a* and *b*. The parameters described by truncated normal distributions are considered unidentifiable based on the time points available for fitting (refer to Supplementary Fig. 8 for identifiability). To supplement the model inference, a more informed prior is provided with an expected mean and deviation. The values for the diffusivity parameter come from previous studies on the potential mean and truncation points of breast tumors^23,52^. Similarly, literature provides the mean and bounds for forming the truncated normal for Adriamycin^69,70^ and cyclophosphamide^71,72^. The standard deviation for all priors based on truncated normal distributions is determined so that the bounds, *a* and *b*, are two deviations away from the mean. The parameters represented by uniform distributions are considered identifiable in this setting, allowing a less informed prior to be utilized. The bounds of the uniform prior for the drug efficacy are provided by literature estimates^23^. After the Karhunen-Loeve decomposition, the *k*_*r*_ parameter is defined in a reduced space, so the desired bounds ([1e-6, 0.1]^23^) cannot be directly applied to each coefficient for this parameter. Instead, an estimate is made of *k*_*r,i,min*_ and *k*_*r,i,max*_ for each coefficient that defines the uniform distributions.

The ABC is implemented using the *pyABC* package in Python^73^. The measured data from V1 serves as the initial condition and the distance is evaluated at V2. The posterior distribution is characterized by a collection of sampled parameter sets, *θ*_*ABC*_, containing 500 independent samples of *θ*, with the complete set comprising the calibrated digital state of the digital twin.

To facilitate rapid simulations when adjusting parameter values during the digital twin calibration, a ROM library is built consisting of parameterized operators for a range of potential values. Operators for parameters following an update are acquired through interpolation from this library. This process is outlined in full detail in ref. ^31^.

### Chemotherapy controls

The treatment controls used to define chemotherapy regimens are comprised of the dose and time for each drug delivery, defined here as ***R***. Each treatment regimen ***R*** is captured by a set of *τ*_*j,k*_ and *C*_*j,k*_ seen in Eq. (3) and can be visualized in Panel B of Fig. 1. The SOC is implemented as ***τ***_***soc***_ = [*τ*_*1*_*, τ*_*2*_*, τ*_*3*_*, τ*_*4*_] for both drug types, where *τ*_*i*_ refers to the day each cycle was delivered. ***C***_***soc***_ represents the standard dosing schedule equal to[1, 1, 1, 1] where the values are a dimensionless, normalized dose (referred to as normalized units (NU) in the subsequent sections) based on the maximum tolerable dose (MTD) of each drug (60 mg/m^2^ for Adriamycin, 600 mg/m^2^ for cyclophosphamide). The vectors ***C***_***soc***_ and ***τ***_***soc***_ define the SOC treatment regimen ***R***_***soc***_. The digital twin is constructed after the first two cycles of A/C are delivered; this results in the optimized regimens retaining the information from the first two cycles, so that ***τ***_***j***_ = [*τ*_*1*_*, τ*_*2*_, $\mathop{{\tau }_{j}}\limits^{ \rightharpoonup }$] and ***C***_***j***_ = [1, 1, $\mathop{Cj}\limits^{ \rightharpoonup }$], where $\mathop{{\tau }_{j}}\limits^{ \rightharpoonup }$ and $\mathop{{C}_{j}}\limits^{\rightharpoonup }$ are vectors containing the times and dosages from the optimization. In clinical practice, the two drugs are delivered at the same time so that ***τ***_***A***_ = ***τ***_***C***_ and ***C***_***A***_ = ***C***_***C***_.

The potential days for chemotherapy deliveries during optimization are determined based on the drug schedule the patient received for cycles 3 and 4, allowing for direct comparison to the SOC simulations. The optimized regimen is allowed to begin on the same day as cycle 3 (i.e., at day of *τ*_*3*_). The time between *τ*_*3*_ and *τ*_*4*_ is defined as Δ*τ*. This time step provides bounds of the potential treatment regimen as well as the end time for the simulation so that $\mathop{{\tau }_{j}}\limits^{ \rightharpoonup }$ = [*τ*_*3*_*,…,τ*_*4*_*,…,τ*_*4*_ + Δ*τ*] ∈ ℕ, where one delivery per day is possible and the optimization is evaluated at *τ*_*4*_ + Δ*τ*. Note this is only possible due to the retrospective study format; in practice the true *τ*_*3*_ and *τ*_*4*_ would not have occurred so Δτ would be calculated from the planned chemotherapy intervals.

The unknown within the optimization problem is the dose vector, $\mathop{{C}_{j}}\limits^{ \rightharpoonup }$, which indicates the chemotherapy concentration delivered at its corresponding time in $\mathop{{\tau }_{j}}\limits^{ \rightharpoonup }$. This vector can have a maximum of 46 variables to optimize depending on the patient’s schedule. To simplify the problem the dose vector is broken into two identical cycles, so that $\mathop{{C}_{j}}\limits^{ \rightharpoonup }$ = [$\mathop{{C}_{x,j}}\limits^{ \rightharpoonup }$, $\mathop{{C}_{x,j}}\limits^{ \rightharpoonup }$], where $\mathop{{C}_{x,j}}\limits^{ \rightharpoonup }$ ∈ [0,1] and has a length of Δ*τ*.

### Optimization problem

There are two main goals of the optimization problem. First, we want to find an optimized treatment regimen that reduces the tumor burden compared to the SOC (P1). Secondly, we aim to find a regimen with a reduced total dose of chemotherapy that provides a not inferior response to the SOC (P2). The treatment controls are adjusted to target the optimization goals of P1 and P2. This is accomplished by minimizing a constrained objective function for each goal, based on predictions from digital twin. For the tumor burden minimization problem (i.e., P1), this results in the following set of equations:

9 $${{\boldsymbol{R}}}_{tumor}^{\ast }\,=\,\mathop{\text{arg}\,\min }\limits_{{\boldsymbol{R}}}\left(\frac{med\left({\int }_{x}N\left({\boldsymbol{R}},{t}_{f},{\theta }_{ABC}\right)\right)}{med\left({\int }_{x}N\left({{\boldsymbol{R}}}_{{\boldsymbol{s}}{\boldsymbol{o}}{\boldsymbol{c}}},{t}_{f},{\theta }_{ABC}\right)\right)}+\frac{\max \left(med\left({\int }_{x}N\left({\boldsymbol{R}},t,{\theta }_{ABC}\right)\right)\right)}{\max \left(med\left({\int }_{x}N\left({{\boldsymbol{R}}}_{{\boldsymbol{s}}{\boldsymbol{o}}{\boldsymbol{c}}},t,{\theta }_{ABC}\right)\right)\right)}\right)$$

subject to:

10 $$\begin{array}{cc}{\Vert {{\boldsymbol{C}}}_{{\boldsymbol{j}}}\Vert }_{1}\le {\Vert {{\boldsymbol{C}}}_{{\boldsymbol{s}}{\boldsymbol{o}}{\boldsymbol{c}}}\Vert }_{1} & \forall j\end{array}$$

11 $$\begin{array}{cc}{\mathcal{M}}({{\boldsymbol{C}}}_{{\boldsymbol{j}}},{{\boldsymbol{\tau }}}_{{\boldsymbol{j}}},{\beta }_{j})\le {\mathcal{M}}({{\boldsymbol{C}}}_{{\boldsymbol{s}}{\boldsymbol{o}}{\boldsymbol{c}}},{{\boldsymbol{\tau }}}_{{\boldsymbol{s}}{\boldsymbol{o}}{\boldsymbol{c}}},{\beta }_{j}) & \forall j\end{array}$$

12 $$\begin{array}{cc}{\mathcal{T}}({{\boldsymbol{C}}}_{{\boldsymbol{j}}},{{\boldsymbol{\tau }}}_{{\boldsymbol{j}}},{\beta }_{j})\le {\mathcal{T}}({{\boldsymbol{C}}}_{{\boldsymbol{s}}{\boldsymbol{o}}{\boldsymbol{c}}},{{\boldsymbol{\tau }}}_{{\boldsymbol{s}}{\boldsymbol{o}}{\boldsymbol{c}}},{\beta }_{j}) & \forall j\end{array}$$

Equation (9) is the objective function, where ${{\boldsymbol{R}}}_{tumor}^{\ast }$ is the treatment regimen that minimizes the tumor burden. The first term of objective function to be minimized is the median cell number at a final time point, *t*_*f*_ (set as *τ*_*4*_ + Δ*τ*, i.e., the end of treatment), and the second term is the maximum value of the median cell time course. The function *med*() indicates to take the median of the distribution. The normalization by the SOC expressions in the denominator of both objective terms guarantees equal weighting between the final tumor burden and the maximum cells during the minimization procedure. Despite being focused only on tumor burden reduction, inclusion of the maximum cells term prevents the optimization from delaying treatment until directly before *t*_*f*_. Equation (10) represents the constraint on the total drug concentration delivered, where ${\Vert \cdot \Vert }_{1}$ denotes the summation of the absolute vector. Equation (11) defines the constraint on the maximum drug concentration at any given time, informed by the functional in Eq. (13):

13 $${\mathcal{M}}(D,\tau ,\beta )=\,\max \left(med\left(\mathop{\sum }\limits_{k}^{T}{C}_{k}{e}^{-\beta (t-{\tau }_{k})H(t-{\tau }_{k})}\right)\right)$$

Equation (12) represents a constraint based on a toxicity estimate from a given treatment. This estimate is determined by Eq. (14):

14 $${\mathcal{T}}(D,\tau ,\beta )={\int }_{\lambda =0}^{\lambda =1.0}med\left(t:\mathop{\sum }\limits_{k}^{T}{C}_{k}{e}^{-\beta (t-{\tau }_{k})H(t-{\tau }_{k})} > \lambda \right)d\lambda$$

where *λ* represents a minimum threshold that is considered toxic, and the term inside the integral defines the time during which the concentration is above this threshold. This function serves to approximate the toxicity based on the assumption that concentrations greater than the LD_50_ (i.e., the concentration which kills 50% of healthy cells) should be avoided. Providing *λ* as an unknown parameter represents the fact that the LD_50_ for each patient is unknown. The integral in Eq. (14) results in units of days·NU (or TU). By constraining the optimized protocols to values obtained by the SOC, we can assume that the outputs will, at worst, provide a risk equivalent to what the patient actually received.

The second optimization problem, P2, is described by the objective in Eq. (15):

15 $${{\boldsymbol{R}}}_{dose}^{\ast }\,=\,\mathop{\text{arg}\,\min }\limits_{{\boldsymbol{R}}}\left(\frac{\sum _{j}{\Vert {{\boldsymbol{C}}}_{{\boldsymbol{j}}}\Vert }_{1}}{\sum _{j}{\Vert {{\boldsymbol{C}}}_{{\boldsymbol{s}}{\boldsymbol{o}}{\boldsymbol{c}}}\Vert }_{1}}+\frac{\max (med({\int }_{x}N({\boldsymbol{R}},t,{\theta }_{ABC})))}{\max (med({\int }_{x}N({{\boldsymbol{R}}}_{{\boldsymbol{s}}{\boldsymbol{o}}{\boldsymbol{c}}},t,{\theta }_{ABC})))}\right)$$

subject to the constraints in Eqs. (11) and (12), with additional constraint:

16 $$med\left({\int }_{x}N\left({\boldsymbol{R}},{t}_{f},{\theta }_{ABC}\right)\right)\le med\left({\int }_{x}N\left({{\boldsymbol{R}}}_{{\boldsymbol{s}}{\boldsymbol{o}}{\boldsymbol{c}}},{t}_{f},{\theta }_{ABC}\right)\right)$$

Equation (15) now represents an equal weighting of the total dose delivered and the maximum cell number over the time course, where ${{\boldsymbol{R}}}_{dose}^{\ast }$ is the treatment regimen that minimizes the dose. The cell number at *t*_*f*_ is now included as a constraint with Eq. (16), where the median cell number from the optimized treatment cannot be greater than the median final cells from the SOC. The problem is still constrained by the maximum concentration (Eq. (11)) and toxicity estimate (Eq. (12)) from each drug.

For both problems the minimize function in Python’s *SciPy* package^74^ is used to solve for the optimal treatment regimen. The basin-hopping algorithm^75^ provides a multi-start wrapper for the COBYLA solver^76^. The speed ups provided by the ROM from Section “ROM derivation” allow access to fast evaluation of objective functions and optimization.

### Statistical analysis

The accuracy of the calibrated digital state is evaluated based on comparisons to measured data at V2 and V3. For individual patients we compute the relative median errors (and ranges) in cell numbers and volumes between the simulated and measured data at these time points, as well as ranges including the simulation uncertainty. The convergence of the ABC sampler is evaluated based on relative changes in metrics describing the parameter distributions. At the cohort level we evaluate concordance correlation coefficients (CCC), which describes the correlation with a penalty for deviation away from the line of unity. The CCC is calculated for both cell numbers and volumes at each time point, with V2 results relating to the calibration accuracy, and V3 results describing the prediction accuracy. As each patient has multiple samples describing the cell and volume distributions, 10,000 random samples are drawn with the CCC calculated for each draw.

The risk (from a clinical perspective) associated with each regimen is based on the total dose delivered, the maximum concentration at any time, and the estimation of toxicity described by Eq. (15); the risk is assessed for each drug independently. The total dose is based directly on the dose delivered at each cycle, with no uncertainty due to model calibration, and is compared directly as a percent difference between the SOC and an alternative regimen. The parameter samples, *θ*_*ABC*_, inform distributions of both max concentration and toxicity estimates for each drug. During the optimization sequence, the constraints in Eqs. (12) and (13) are evaluated by taking the median value of the max concentration and toxicity estimate, respectively. After optimization, the distributions of max concentration and toxicity from the SOC and optimized regimen are compared using Mann-Whitney *U*-Tests to check for significant differences.

After optimization we compare the tumor response achieved by the SOC to that achieved with the new treatment regimen obtained from the optimization problems independently. For individual patients we compare the distributions output from the objective and constraint functions (with median function removed) to quantify the benefit and risk of the optimized treatment. We evaluate the percent change in tumor burden (both cell number and volumes) between the two regimens and use Mann-Whitney *U*-tests to test for significant differences in total cell number, relative change in cell number, total volume, relative change in volume, total dosage, max drug concentration, and toxicity estimations. The quantities of interest associated with risk are calculated from the drug time course starting with cycle three, as a direct comparison to when the optimization starts. To assess performance across the cohort we compare median and ranges of the percent changes in total and max cell number, total and max volume, total chemotherapy dosages, max drug concentration, and toxicity estimations. In Section “Determining factors for optimization reward” we compare optimization results with patients grouped by clinical response classification (pCR vs. non-pCR). We compare quantities of interest of both groups with *U*-tests, and present optimization results against parameter fits using a bootstrapping method to account for uncertainty, with 10,000 random samples drawn from the parameter and optimization distributions. This method provides a mean, and confidence interval for linear regression models based on calibrated parameter values, with significance evaluated by Pearson correlation coefficients.

## Supplementary information

Supplemental Material

## Data Availability

All patient data used in this study is confidential and not available for public use. Example patients have been created and added to a repository to supplement this. The entire framework is available under an MIT license at https://github.com/cchristenson2/TNBC_DigitalTwins.

## Code availability

The entire framework is available under an MIT license at https://github.com/cchristenson2/TNBC_DigitalTwins.
