# Supplementary material for: Personalizing neoadjuvant chemotherapy regimens for triple-negative breast cancer using a biology-based digital twin
Source: NPJ Syst Biol Appl. 2025 May 23;11:53. doi: 10.1038/s41540-025-00531-z (PMC12102339; doi:10.1038/s41540-025-00531-z)
Supplement: Supplementary file 1 — Supplemental Material [file 41540_2025_531_MOESM1_ESM.pdf]

Supplemental Material for: Personalizing chemotherapy regimens for triple-negative breast cancer patients using a biology-based digital twin

Chase Christenson, Chengyue Wu, David A. Hormuth II, Anirban Chaudhuri, Jingfei Ma, Gaiane M. Rauch, Thomas E. Yankeelov.

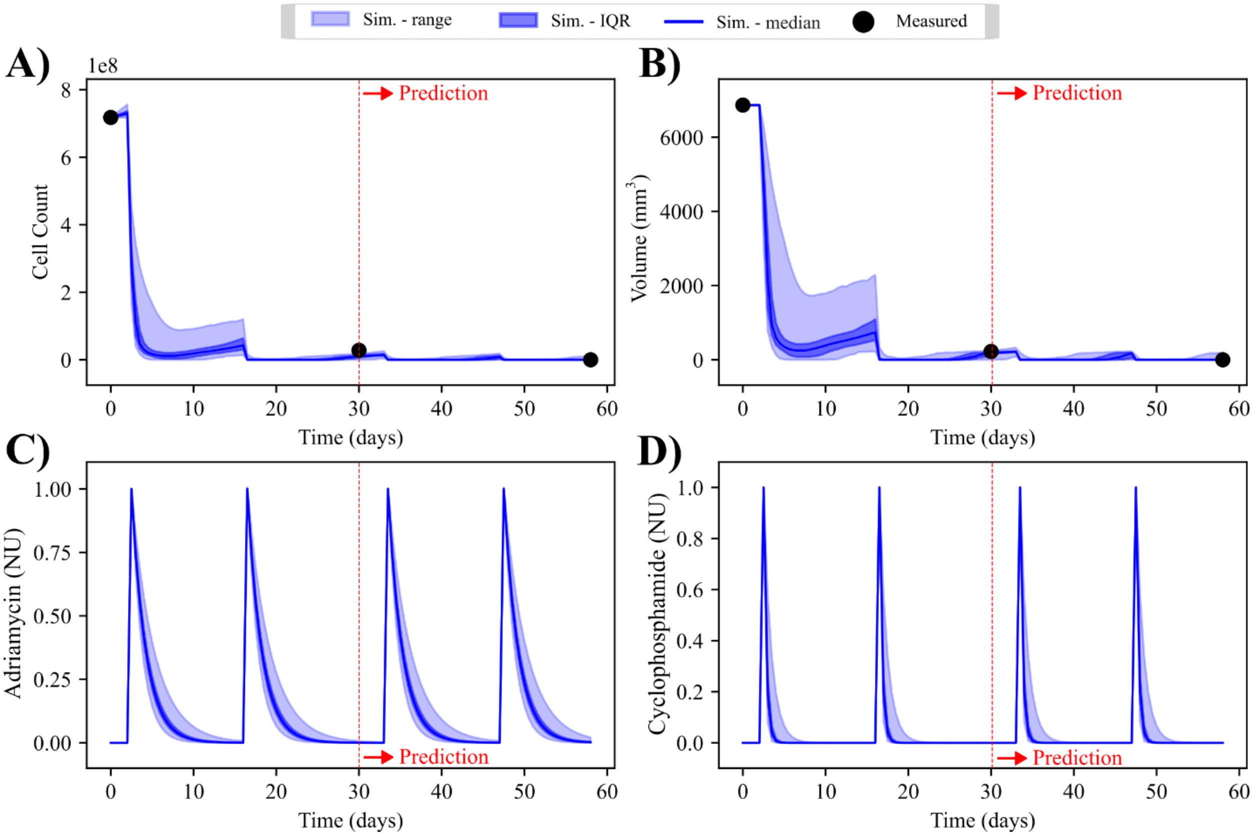

Supplementary Figure 1. High performing patient with approximate Bayesian calibration. Panels A and B provide the cell and volume time courses from the calibrated digital twin, where the prediction phase starts at 30 days. These plots show high accuracy between the simulations and measured data at both V2 and V3. The median percent error of the simulated cells is equal to -2.58% (-3.10%, 2.17%) at V2 and 0.00% (0.00%, 0.06%) at V3. For the changes in volumes, the median percent error is -0.54% (-1.35%, -0.27%) at V2 and 0.00% (0.00%, 0.13%) at V3. Panels C and D show the drug concentration curves for Adriamycin and cyclophosphamide, respectively.

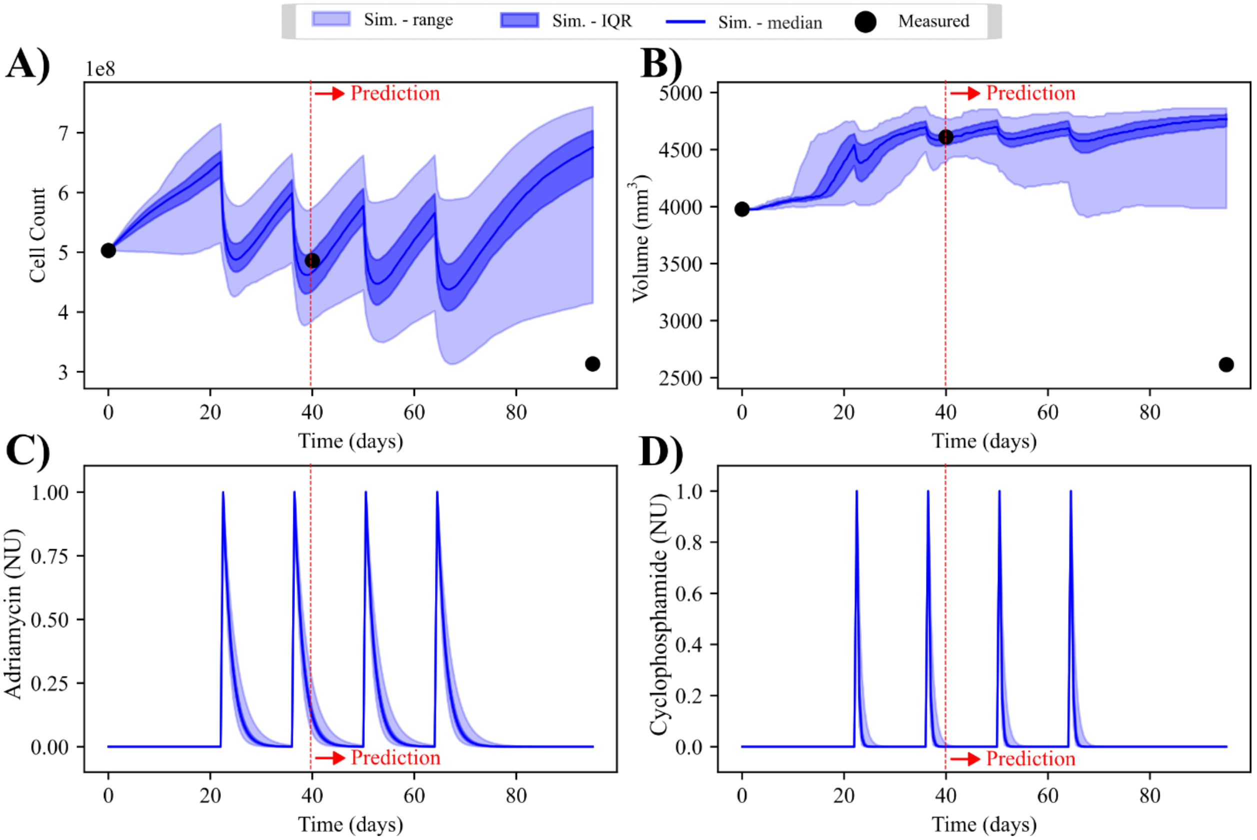

Supplementary Figure 2. Poor performing patient with approximate Bayesian calibration. Panels A and B provide the cell and volume time courses from the calibrated digital twin, where the prediction phase starts at 40 days. These plots show high accuracy at V2 with a median percent error for cells of -3.94% (-10.50%, 3.20%) and -0.38% (-1.51%, 0.75%) for volumes. These values increase significantly ($p < 0.001$) at the V3 prediction, with a median percent error in cells of 115.70% (99.96%, 124.58%) and 82.39% (79.98%, 84.05%) for volumes. Panels C and D show the drug concentration curves for Adriamycin and cyclophosphamide, respectively.

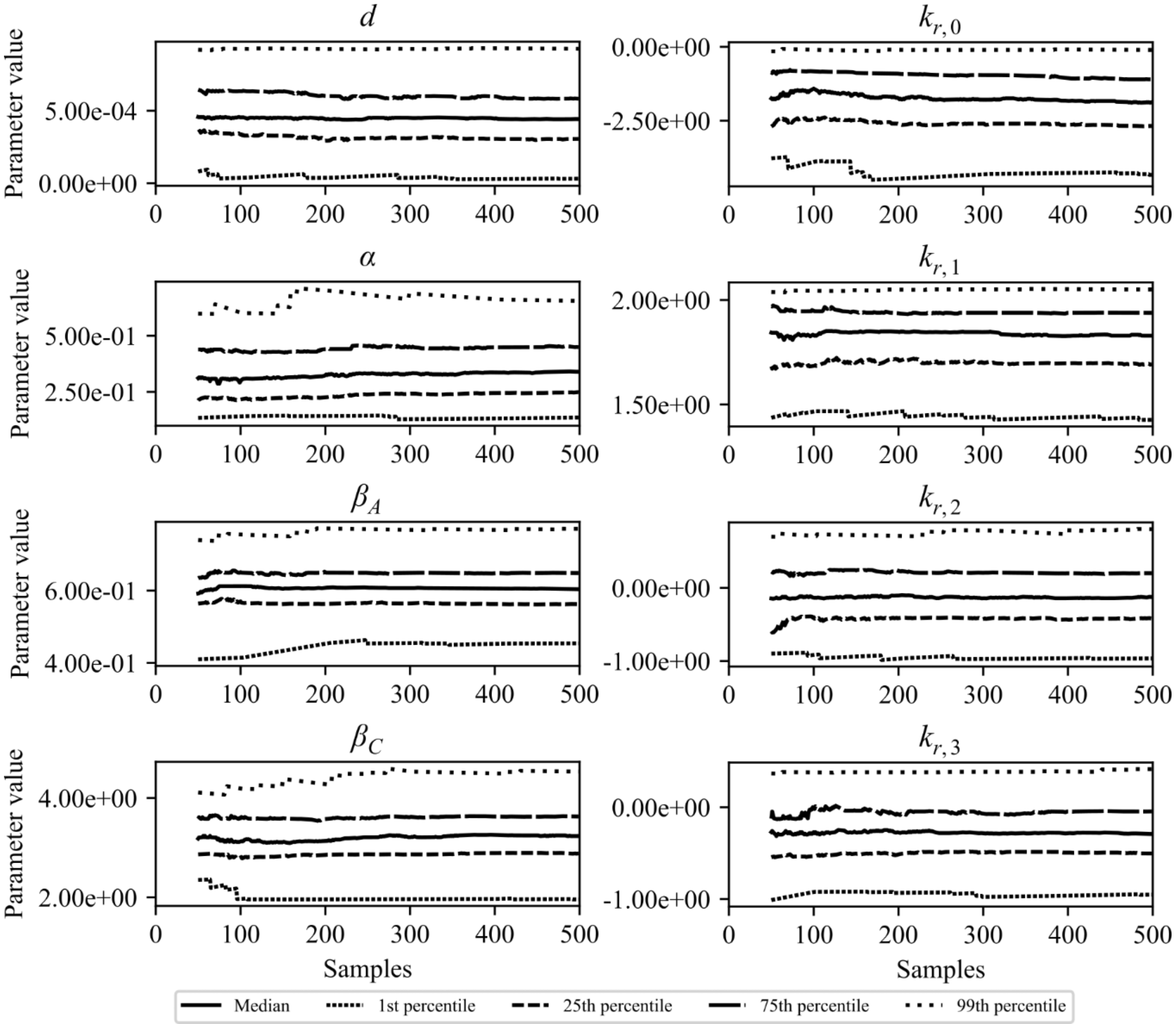

Supplementary Figure 3. Convergence of parameters from ABC sampler. Plots of distribution of metrics for each parameter based on sample number are displayed. In each axes, the first ~200 samples display large variation in all metrics but reach convergence by the 500 sample mark. There is an increase in variation at the quartiles with a maximum relative change of 0.50% and 1.11% for the 25th and 75th percentile, respectively. The tails are relatively stable with a change maximum change of 0.21% and 0.38% for the 1st and 99th percentile respectively. A converged distribution is critical for ensuring outputs from probabilistic simulations are representative of the true distribution.

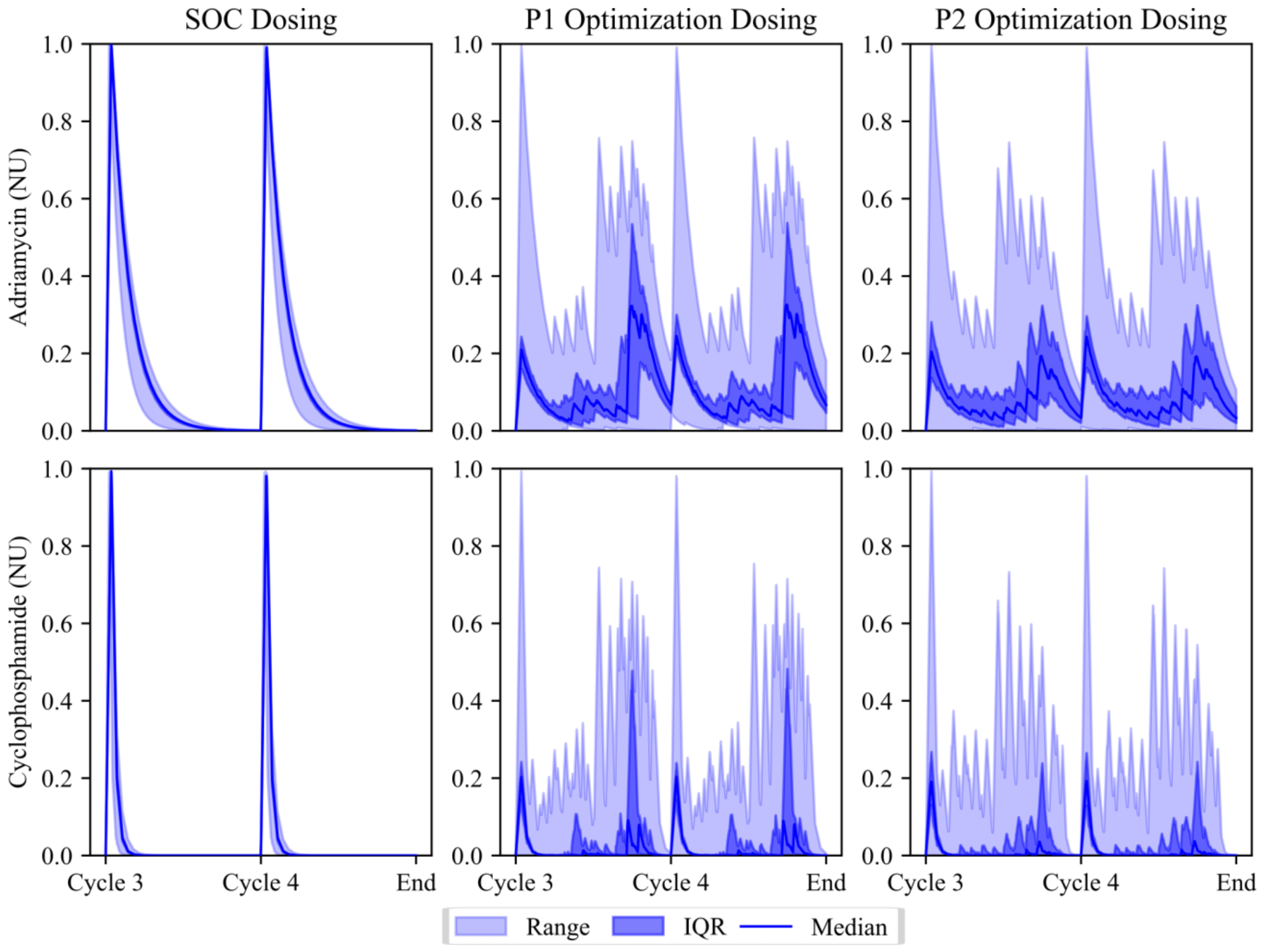

Supplementary Figure 4. Delivery schedules averaged across the population. For each plot we present the median, interquartile range, and range of the dose schedule normalized in the temporal domain. The top row provides the drug concentrations for Adriamycin from the standard of care (left), cell minimization problem, P1 (middle), and dose minimization problem P2 (right). The bottom provides the same plots for cyclophosphamide. These plots show how the drug is delivered on average depending on how the schedule is chosen, using either the standard of care, cell minimization, or dose minimization.

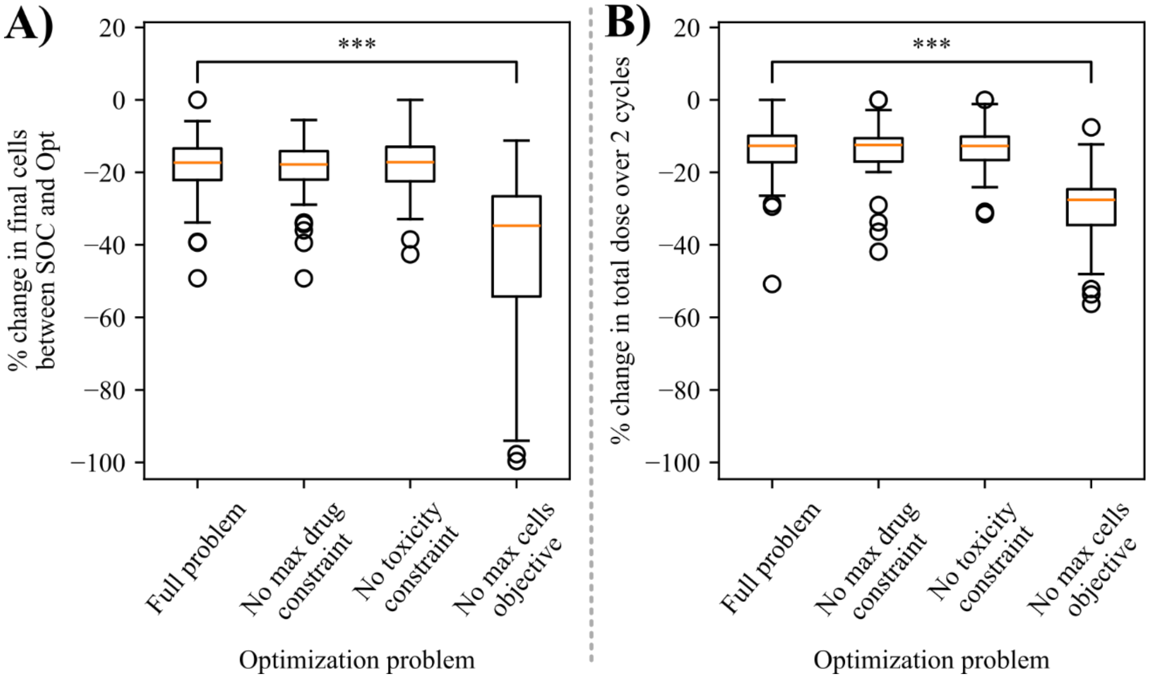

Supplementary Figure 5. Optimization scenarios with individual constraints or weightings removed. Panel A provides the P1 optimization results for the full problem compared with optimizations after removing one of the max drug concentration constraint, toxicity constraint, or maximum cell number objective weighting. When compared with full model, only removing the max cell weighting results in a significant difference across the cohort (p-value < 0.001) with the no weighting problem resulting in a better percent change in final cells when comparing optimized and standard of care regimens. Similarly, for the P2 optimization in panel B, the only significant difference compared to the full optimization problem when removing model terms is from the removal of the max cells weighting (p-value < 0.001). These results highlight that constraining the problem in different ways can change optimal outputs.

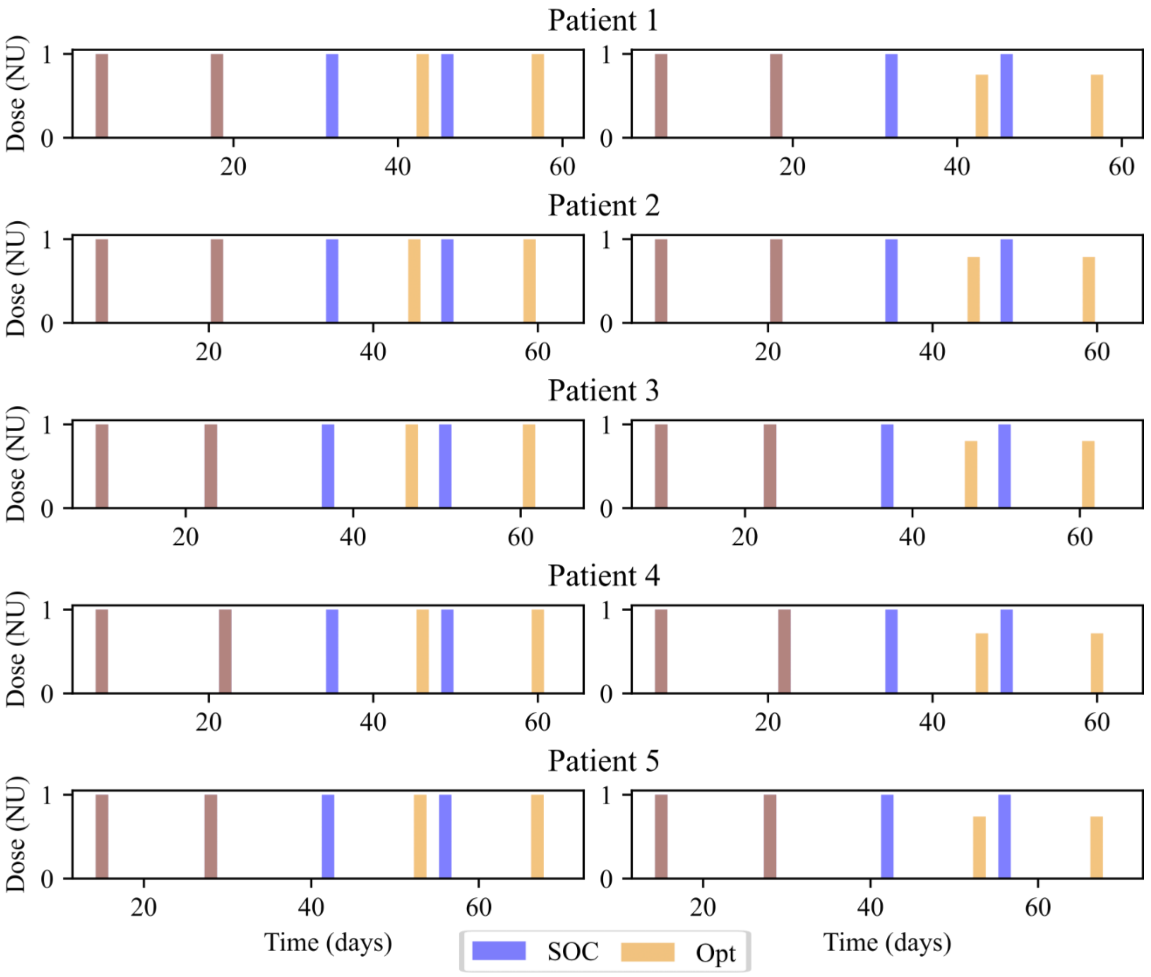

Supplementary Figure 6. Example regimens from optimization problem with no maximum cells weighting in objective. For the first 5 patients, regimens from the P1 optimization (left column) and P2 optimization (right column) are shown compared to the SOC when the max cells term is removed from the objective function. In all cases shown (as well as all other patients), the optimized protocol favors a delayed treatment where the optimized delivery is near the end of cycles three and four, rather than on the first day as in the SOC. The optimized protocols for both P1 and P2 result in a median time between cycles two and three of 25 days, compared to a median of 14 days with the SOC regimens.

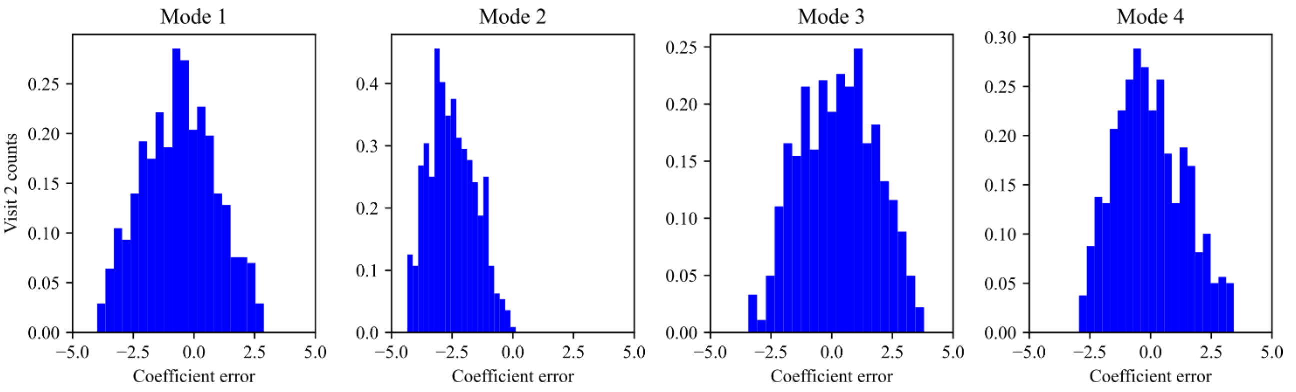

Supplementary Figure 7. Model errors for each mode. The overall distribution of model errors for the reduced coefficients are provided for each mode. These values are reflective of the difference between simulations and measured data at the visit 2 time point. Error distributions from each respective mode are significantly different from the others with p-values < 2×10^{-3}. These differences prevent fitting a single distribution that represents all modal errors.

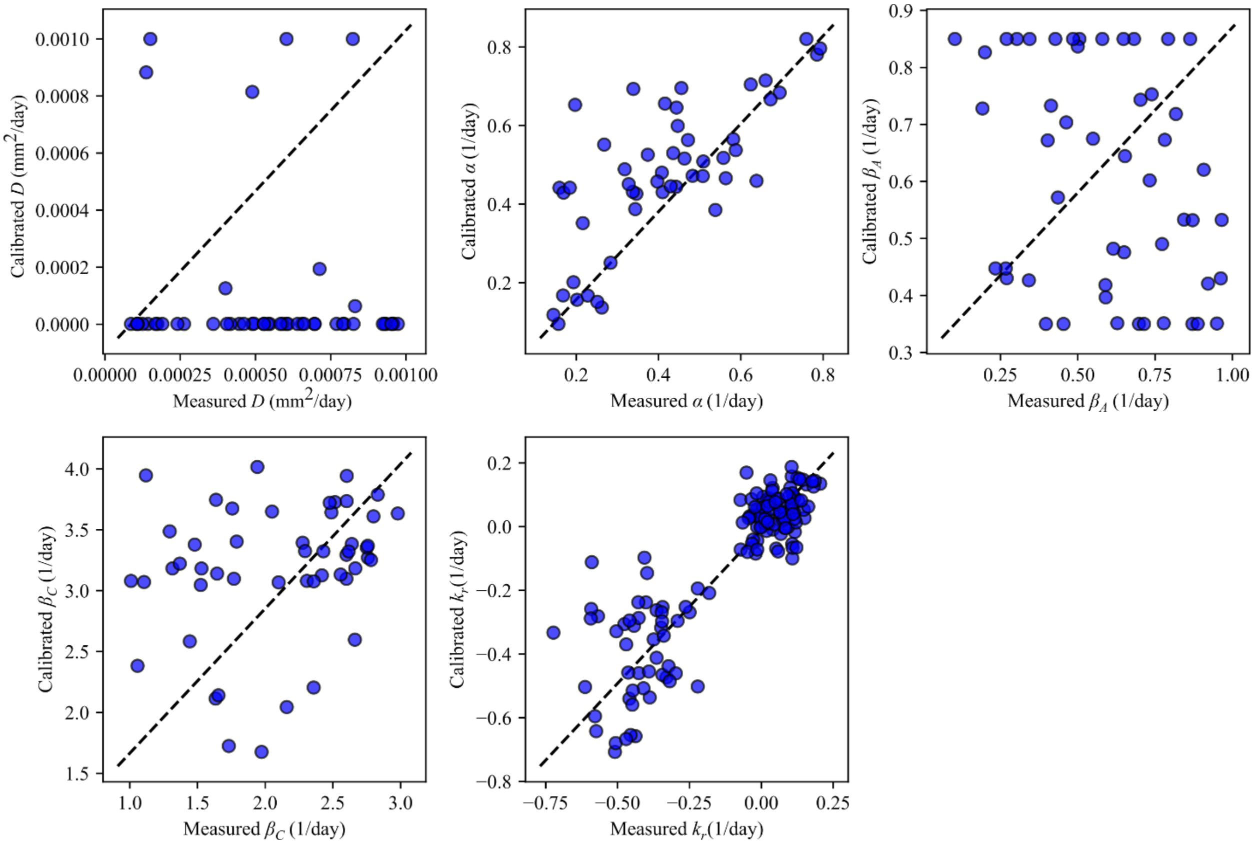

Supplementary Figure 8. Parameter identifiability using in silico data. Results are presented comparing parameter estimations from a calibrated model to true values used to

generate in silico data. These 50 data points were generated in MATLAB with an SNR of 30. The dotted line of unity in each plot represents the ideal line of distribution, with deviations away being unwanted. Only the treatment efficacy (α), and proliferation (k_r) show high correlation, with CCC = 0.70 and 0.88, respectively, whereas diffusivity (D), and decay rates (β_i), show low correlation with CCC = 0.09 and 0.29, respectively.
